# Integrated analysis of metabolic gene features and the immune microenvironment: identification of DPYD-mediated prognostic model and therapeutic targets in pancreatic cancer

**DOI:** 10.1186/s12876-026-04726-4

**Published:** 2026-03-14

**Authors:** Haoyu Bao, Bowen Pu, Shengpeng Zhu, Yongqiang Chen, Xiaohong Wang, Weihao Guo, Qikai Sun, Yeben Qian

**Affiliations:** Department of General surgery, the First Affiliated Hospital of Anhui Medical University, 218 Jixi Road, Hefei, 230022 China

**Keywords:** Pancreatic Ductal Adenocarcinoma, DPYD, Metabolic Reprogramming, Machine Learning

## Abstract

**Background:**

Pancreatic ductal adenocarcinoma (PAAD) is a malignant digestive system tumor characterized by extremely poor prognosis and significant molecular heterogeneity. Metabolic reprogramming, a key hallmark of cancer, is considered a vital mechanism underlying immune evasion and malignant progression in pancreatic cancer.

**Methods:**

RNA-sequencing data and clinical information of PAAD patients were retrieved from TCGA and GTEx datasets. Metabolism-related genes were curated from previous literature (*n* = 712). Differential expression analysis between TCGA-PAAD tumors and GTEx normal pancreas identified 681 metabolism-associated differentially expressed genes (MAGDEGs), from which 19 prognosis-related metabolism genes (MAGCOXs) were further screened using univariate Cox regression. Consensus clustering determined metabolic subtypes. A prognostic model was constructed using StepCox [forward] + GBM algorithm. Kaplan–Meier survival curves, log-rank test, and ROC curves validated predictive performance. Differences in immune infiltration, TMB, IPS, TIDE, and anticancer drug sensitivity related to risk scores were analyzed. Single-cell clustering and pseudotime analyses further elucidated the significance of metabolism-related genes. DPYD expression in PAAD tissues and cell lines was assessed via qRT-PCR, WB, and IHC. Functional assays and proteomic analysis were used to explore the role of DPYD in PAAD.

**Results:**

The model reliably distinguished high- and low-risk patient groups, demonstrating robust predictive stability and generalizability across independent GEO validation cohorts. The high-risk group showed enhanced immune evasion characteristics and distinct drug sensitivity profiles. DPYD overexpression correlated with poor prognosis; DPYD knockdown inhibited colony formation and migration in BxPC-3 cells, whereas its overexpression promoted these capacities. Proteomics analysis revealed DPYD deficiency disrupted key metabolic pathways including fatty acid metabolism, TCA cycle, and amino acid metabolism. Enrichment analysis indicated DPYD involvement in pancreatic cancer and related diseases.

**Conclusion:**

We identified metabolism-associated DEGs and further screened 19 prognosis-related metabolism genes, based on which a metabolism-associated prognostic model with excellent prognostic value for PAAD patients was constructed. The high-risk group displayed stronger immune evasion characteristics and distinct drug sensitivity compared to the low-risk group. External validation confirmed the model’s predictive stability and generalizability. DPYD was predominantly enriched in macrophages and T-cell subsets, and its high expression positively correlated with poor prognosis. Elevated DPYD expression was validated in PAAD tissues and cell lines, and functional assays demonstrated that DPYD knockdown inhibited tumor cell clonogenicity, migration, and disrupted key metabolic pathways.

**Supplementary Information:**

The online version contains supplementary material available at 10.1186/s12876-026-04726-4.

## Introduction

Pancreatic ductal adenocarcinoma (PAAD) is one of the most lethal malignancies of the digestive tract worldwide. It is characterized by a subtle clinical onset, aggressive progression, and the absence of effective methods for early detection [1, 2]. It exhibits high resistance to chemotherapy, radiotherapy, and targeted therapies, with a five-year overall survival (OS) rate of less than 10% [3, 4]. This extremely poor outcome underscores the urgent need for early-stage detection and risk stratification strategies in pancreatic cancer [5, 6]. While immunotherapy has shown promising efficacy in various cancers, such as melanoma and non-small cell lung cancer in recent years, its clinical efficacy in PAAD remains limited. This phenomenon is primarily attributed to the highly fibrotic microenvironment and immunosuppressive state of the tumor [7–9], as well as its low tumor mutation burden and complex metabolic adaptability [10].

Metabolic reprogramming is a critical hallmark of tumorigenesis and progression [11, 12]. Notably, metabolic reprogramming comprises various metabolic pathways, including glucose, lipid, amino acid, and nucleotide metabolisms, as well as redox homeostasis. Through metabolic regulation, tumor cells utilize and synthesize both energy and biomaterials to meet their proliferative and survival requirements, thereby facilitating their unlimited proliferation and migration under certain conditions. Moreover, the tumor immune microenvironment can be epigenetically modulated through aberrant expression of metabolism-related regulatory genes [13, 14], thereby influencing immune cell differentiation, infiltration, and antitumor functions, which may contribute to an immune-evasive phenotype.

Research indicate that certain metabolic factors (e.g., LDHA, PKM2) can serve as prognostic indicators for patient with pancreatic cancer. However, existing studies fail to provide comprehensive identification of cancer subtype and theoretical framework for the construction of a corresponding risk model from a metabolic perspective. Additionally, existing studies are faced by a myriad of key shortcomings, including difficulty in conducting external validation across different datasets, integrated analyses with immunosuppressive states, functional assays of key metabolic genes, and assessment of drug sensitivity [15].

In this study, an integrated analysis of the TCGA and GTEx datasets was conducted to screen 19 prognosis-related metabolism genes (MAGCOXs) closely associated with survival outcomes in patients with pancreatic ductal adenocarcinoma (PAAD). Metabolic subtypes of pancreatic cancer were delineated using consensus clustering, followed by the development of a metabolic prognostic scoring model using the Stepwise Cox regression (forward) in combination with GBM algorithm. Multi-platform adaptability validation was performed within and across GEO validation cohorts. The potential relationship between model scores and immune phenotypes was systematically investigated by integrating immune infiltration analysis and immunotherapy predictive indicators such as IPS, TMB, and TIDE. Focusing on DPYD—the core gene of the model—its correlation with sensitivity to various anticancer agents was assessed using data from the CellMiner database. Additionally, single-cell RNA-sequencing (scRNA-seq) analysis was used to reveal DPYD distribution across different cellular populations. Further, in vitro functional assays and proteomics analysis were employed to validate the role of DPYD in regulating pancreatic cancer cell proliferation, migration, and metabolic pathways.

This study follows a systematic, closed-loop research framework integrating bioinformatics prediction, immunometabolic correlation, and experimental validation, collectively identifying *DPYD* as a central regulator linking metabolic and immune pathways to PAAD. Notably, DPYD may serve as a novel biomarker and therapeutic target in pancreatic cancer, providing theoretical support for precision medicine in oncology. Consequently, metabolism-based research in combination with molecular subtyping and identification of key metabolic drivers in pancreatic cancer, hold significant potential in elucidating the underlying molecular mechanisms of pancreatic cancer and facilitates the development of precision therapy.

## Materials and methods

### Data collection and processing

Transcriptomic data and corresponding clinical information of pancreatic ductal adenocarcinoma (PAAD) patients were obtained from The Cancer Genome Atlas (TCGA), and normal pancreatic tissue expression data were obtained from the Genotype-Tissue Expression (GTEx) project. All data were downloaded using the R packages “TCGAbiolinks” and “recount” [16]. For downstream analyses, gene identifiers were harmonized across datasets, and expression matrices were transformed to a comparable scale (log2-transformed normalized expression) before statistical analysis. Given that TCGA and GTEx originate from different projects and processing pipelines, residual cross-platform technical variability may persist; therefore, TCGA–GTEx differential expression findings were interpreted cautiously and are discussed as a limitation. A set of metabolism-related genes was curated based on previously published literature, comprising 712 candidate genes [17]. In addition, GSE28735 and GSE57495 were included as external validation cohorts to evaluate the robustness and generalizability of the prognostic model across independent datasets.

### Differential expression analysis and prognostic gene screening

The R package “limma” was used to integrate and normalize transcriptomic profiles from the TCGA-PAAD and GTEx datasets [18], followed by differential expression analysis. No cross-dataset batch correction (e.g., ComBat) was applied prior to TCGA–GTEx differential expression analysis; this limitation is discussed in the Discussion. The screening criteria were set as FDR < 0.005 and |log_2_FC| > 1. Subsequently, univariate Cox proportional hazards regression analysis was performed on the differentially expressed metabolism-related genes to identify prognostically significant MAGCOXs in combination with clinical survival data. Additionally, LASSO Cox regression was applied within the ML.Dev.Prog.Sig framework (Mime1 package), where cross-validation-based penalty selection was internally performed according to the default implementation of the package. The random seed was fixed (seed = 123) to ensure computational reproducibility.

To provide an overview of the analytical framework, metabolism-related genes were first curated from previously published literature (*n* = 712). Differential expression analysis between TCGA-PAAD tumor samples and GTEx normal pancreatic tissues identified metabolism-associated differentially expressed genes (MAGDEGs), which were subsequently subjected to univariate Cox proportional hazards regression to obtain prognosis-related candidate genes (MAGCOXs). In addition, LASSO regression with cross-validation was applied as an auxiliary feature-reduction step to improve the robustness of candidate gene screening. These candidate genes were then incorporated into a machine learning–based survival modeling framework for final model construction and external validation, as described in Sect.  2.4. The complete workflow is summarized in Supplementary Table S13.

### Consensus clustering analysis

Unsupervised clustering of MAGCOX expression patterns was conducted using the “ConsensusClusterPlus” package [19]. A total of 1,000 resampling iterations were performed to evaluate the stability of clustering solutions, with the number of cluster (k) ranging from k = 2 to k = 10. The cumulative distribution function (CDF) and the delta area curve were used to determine the optimal number of clusters. Ultimately, k = 2 was selected as the classification basis for metabolic subtypes. Principal component analysis (PCA) and heatmaps were subsequently used to validate expression differences and associations with clinical features between subtypes.

### Prognostic model construction and validation

MAGCOXs expression profile was used as feature variables to construct 101 unique combinations of machine learning algorithms for model construction. Each algorithm combination was evaluated using repeated bootstrap resampling within the TCGA cohort. Briefly, for each bootstrap iteration (*n* = 1000), samples were resampled with replacement to fit the model, and predictive performance was assessed by Harrell’s concordance index (C-index) on the out-of-bag (non-resampled) samples. A fixed random seed (seed = 123) was used during resampling procedures to ensure reproducibility of model evaluation. The final C-index for each algorithm combination was summarized as the mean (± SD) across bootstrap iterations to reflect both discrimination and stability. To mitigate overfitting arising from extensive model exploration, model selection prioritized algorithm combinations showing consistently high and stable C-index across bootstrap iterations and reproducible performance in the two independent external cohorts (GSE28735 and GSE57495), rather than selecting a model based on a single best-performing resampling result. The same feature set (MAGCOXs) and modeling pipeline were applied across all algorithm combinations to ensure methodological comparability. The R packages “survcomp” and “timeROC” were used to conduct model construction and validation. Following construction of the model in the TCGA training cohort, external validation was conducted using two independent datasets, GSE28735 and GSE57495. Model performance was comprehensively assessed using Kaplan–Meier survival curves analysis, log-rank tests, and time-dependent ROC curves. For Cox regression analyses involving clinical variables and the risk score, proportional hazards assumptions were evaluated using Schoenfeld residuals (cox.zph).

Model selection was based on bootstrap-based Harrell’s concordance index (C-index) performance across the training cohort and external validation cohorts, with preference given to the algorithm combination showing the highest overall discrimination and the most stable performance across datasets. Specifically, all 101 combinations were ranked according to the average C-index across cohorts (TCGA, GSE28735, and GSE57495), with stability (lower SD across bootstrap iterations) used as a secondary criterion to break ties. Based on this criterion, the Stepwise Cox regression (forward) plus Gradient Boosting Machine (GBM) survival model was selected as the final prognostic model. The median risk score was determined within each cohort for dichotomization, and the same risk score generation procedure was consistently applied across all cohorts.

The final prognostic signature comprised six metabolism-associated genes (CA12, CA8, DPYD, INPP4B, MTHFD1, and PTGS2). The model-derived risk score for each patient was generated by the finalized StepCox[forward] + GBM framework using the expression profiles of these six genes, and patients were subsequently stratified into high- and low-risk groups according to the median risk score in each cohort.

Comparison with published prognostic models. To ensure methodological fairness, all 33 published prognostic models were re-implemented using the same normalized expression matrix and identical clinical cohorts as used in the present study. Risk scores for each published model were calculated strictly according to the originally reported gene sets and scoring schemes (coefficients when available) without re-training or re-optimization. Model performance was evaluated using the same statistical framework (C-index and time-dependent ROC) and identical outcome definitions across TCGA and external validation cohorts. No cohort-specific parameter tuning was performed for published models to avoid performance inflation.

### Immune microenvironment and immunotherapy-related analysis

Multiple algorithms, including CIBERSORT-ABS, EPIC, MCPcounter, and TIMER, were employed to comprehensively evaluate the status of immune cell infiltration. ssGSEA was used to assess both immune function scores and pathway activities. The associations between model scores and immunotherapy predictive indicators—IPS, TMB, and TIDE—were investigated to explore the predictive power of the model for immunotherapy response [20].

Immune cell infiltration levels were estimated using established computational deconvolution algorithms based on bulk RNA sequencing data. It should be noted that these approaches infer relative immune cell proportions rather than providing direct quantitative measurements. Therefore, immune infiltration results should be interpreted as computational estimations. No additional experimental validation (e.g., flow cytometry or immunohistochemistry) was performed in the present study.

### Drug sensitivity analysis

Drug sensitivity data related to the NCI-60 cell lines were downloaded from the CellMiner database. Pearson correlation coefficients were calculated to determine the relationship between the expression levels of key genes, including DPYD, and the IC50 values of various anticancer drugs. The “pRRophetic” package was used to estimate IC50 values of common targets and chemotherapeutic agents (such as Dabrafenib, Vorinostat, and Niraparib) in TCGA samples. Additionally, differences in drug responses between model-defined risk groups were evaluated. These drug–gene associations and IC50 predictions are exploratory and hypothesis-generating, because CellMiner is based on pan-cancer NCI-60 cell line pharmacogenomic data and pRRophetic provides in silico estimates; therefore, PAAD-specific experimental and/or clinical validation is required before any therapeutic implications can be inferred.

### Single-cell RNA-seq analysis

Single-cell data from the GSE212966 dataset were downloaded and processed. The Seurat package was used to perform quality control, normalization, PCA, UMAP clustering, and annotation of the cell subtype. Pseudotime trajectory analysis was performed using Monocle2 to determine expression dynamics and developmental pathways of key genes (such as DPYD and PTGS2) across cell types. Tumor and adjacent tissues served as the grouping bases to construct cell density maps and fate trajectories [21].

### Cell culture and transfection

Human pancreatic cancer BxPC-3 cells were cultured in DMEM supplemented with 10% fetal bovine serum (FBS), 100 IU/mL penicillin, and 100 µg/mL streptomycin. The cells were cultured in a humidified incubator maintained at 37 °C with 5% CO₂. Cells with either overexpression or knock-down of DPYD were generated using the jetPRIME transfection reagent (101000046, Polyplus) as per the manufacturer’s protocol. Plasmids pECMV-3xFLAG-DPYD (p3121,MIAOLING PLASMID), hDPYD-shRNA1, and hDPYD-shRNA2 were transfected into HEK-293T cells to facilitate lentiviral packaging and concentration. After 24 h of infection, BxPC-3 cells were subjected to selection with 2 µg/mL puromycin (ST551, Beyotime). Stably transduced cells were subsequently collected for downstream experiments.

### Western blot

Proteins were isolated from cells using RIPA lysis (P0013B, Beyotime)buffer with protease and phosphatase inhibitors (P0038, Beyotime). The BCA assay (P0010, Beyotime) was used to perform protein quantification, then the samples were denatured with a loading buffer (P0015, Beyotime) and heat, separated using SDS-PAGE, and transferred to PVDF membranes (IPVH08100, Millipore). The membranes were blocked with 5% skim milk for 1 h, followed by incubation with DPYD primary antibody (JE54-79,HUABIO) and then rabbit secondary antibody (GB23303, Servicebio). Protein bands were detected using the SuperPico ECL kit (P0018S, Beyotime) and imaged with a Bio-Rad chemiluminescence system.

### Quantitative real-time PCR (qRT-PCR)

Total RNA extraction was conducted using the RNA extraction kit (RC112, Vazyme). The concentration of the extracted RNA was measured using a Thermo Scientific Nanodrop 2000 spectrophotometer. Subsequently, cDNA was synthesized using HiScript III RT SuperMix (R323, Vazyme), followed by performing the qRT-PCR. Relative gene expression levels were determined using the 2^−ΔΔCt^ method, with expression levels normalized to β-Actin, which served as the internal control.

### Immunohistochemistry (IHC)

Following deparaffinization and rehydration of the tissue section, they were then subjected to antigen retrieval in a citrate buffer (G1219-1 L, Servicebio). After blocking with 10% goat serum at room temperature for 1 h, the tissue sections were incubated with DPYD primary antibody (JE54-79, HUABIO) and rabbit secondary antibody (GB23303, Servicebio), followed by DAB staining (ZLI-9017, ZSGB-BIO).

### Colony formation assay

BxPC-3 cells with DPYD overexpression, knockdown, or control were trypsinized during the logarithmic growth phase. They were then centrifuged at 1,000 rpm for 5 min and resuspended in a complete medium. Cells were seeded at 1,000 cells per well in 6-well plates. The cells were cultured for 2–3 weeks with the medium replaced every 3 days, visible colonies were fixed with 4% paraformaldehyde, stained with 1 mL crystal violet for 20 min, washed, air-dried, and the images captured. The number and size of the cell colonies were quantified.

### Wound healing assay

Under a biosafety cabinet, equally spaced lines were drawn on the bottom of 6-well plates using a sterile marker. Cells were seeded to appropriate density and cultured overnight. Upon reaching approximately 90% confluence, a straight scratch was made along the marked lines using a 200 µL pipette tip. After aspiration of the medium and washing with sterile PBS, serum-free medium was added. Initial images were captured under a microscope, and a second round of imaging was performed 24 h later to assess wound closure.

### Quantitative proteomic analysis

Quantitative label-free proteomic analysis was performed in DPYD-knockdown (shDPYD) and control BxPC-3 cells, with three independent biological replicates per group (*n* = 3). Total proteins were extracted, digested with trypsin, and analyzed by liquid chromatography–tandem mass spectrometry (LC–MS/MS).

Raw MS data were processed using MaxQuant software against the UniProt human reference database. The false discovery rate (FDR) was controlled at 1% at both peptide and protein levels.

Protein intensity values were normalized prior to statistical comparison. Differentially expressed proteins (DEPs) between groups were identified using Student’s t-test with Benjamini–Hochberg correction for multiple testing. Proteins with Benjamini–Hochberg adjusted *P* < 0.05 and fold change ≥ 1.2 were considered differentially expressed.

Functional enrichment analyses, including Gene Ontology (GO), Kyoto Encyclopedia of Genes and Genomes (KEGG), and Disease Ontology (DO), were performed based on the identified DEPs.

## Results

### Differential expression and prognostic analysis of metabolism-related genes

**Fig. 1:**
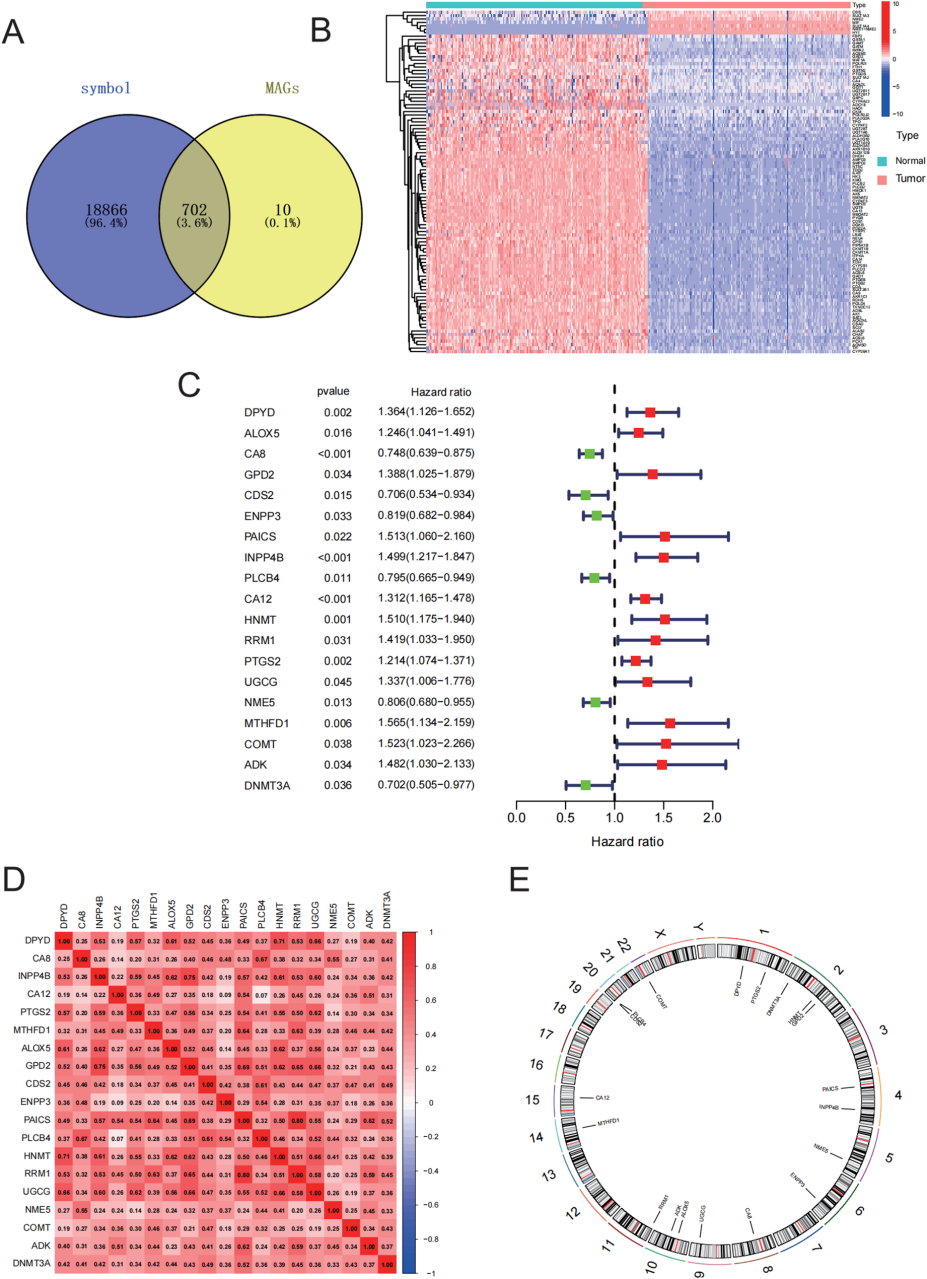
Analysis of the Differential Genes. **A** Venn diagram displaying the intersection between 19,568 genes from the TCGA-GTEx-PAAD dataset and 712 metabolism-associated genes (MAGs). **B** Heatmap showing the MAGDEG expression profiles in the TCGA-GTEx-MAGs dataset. **C** A forest plot for the prognostic relevant genes among MAGDEGs using results of the univariate Cox regression (uniCox) analysis. **D** Correlation heatmap of MAGCOXs in the TCGA dataset. **E** Chromosomal mapping of MAGCOXs

In this study, transcriptomic data of 182 PAAD samples from the TCGA database and 167 normal pancreatic tissue samples from the GTEx database were integrated. Based on metabolism-associated genes (MAGs), differentially expressed genes (DEGs) were identified in the combined TCGA-GTEx dataset (Fig. 1A). The limma package with criteria of |log_2_FC| > 1 and FDR < 0.005 was used to perform differential expression analysis, resulting in the identification of a total of 681 MAGDEGs. Their expression patterns were visualized using a heatmap (Fig. 1B), with genes such as DPYD (downregulated) and PAICS (upregulated) exhibiting significant differences in tumor tissues.

Out of the 681 MAGDEGs that were significantly associated with the prognosis of pancreatic cancer (*p* < 0.05), 19 metabolism-associated genes (MAGCOXs) were identified using univariate Cox regression analysis. A forest plot (Fig. 1C) was used to illustrate the hazard ratios (HR) of key genes, including oncogenic risk-related genes: DPYD (HR = 1.364, *p* = 0.0002), PAICS (HR = 1.510, *p* = 0.002); protective genes: CA8 (HR = 0.748, *p* < 0.001), ENPP3 (HR = 0.819, *p* = 0.003). These genes were found to be involved in nucleotide metabolism, suggesting a multidimensional association between metabolic imbalance and tumor progression.

Gene co-expression network analysis (Fig. 1D) further revealed synergistic regulatory relationships among the MAGCOXs. Chromosomal mapping (Fig. 1E) indicated significant clustering of the 19 MAGCOXs on chromosomes 1 (DPYD, PTGS2), 2 (DNMT3A, HNMT, GPD2), and 4 (PAICS, INPP4B), which may be associated with genomic instability or copy number variations in these regions.

**Fig. 2:**
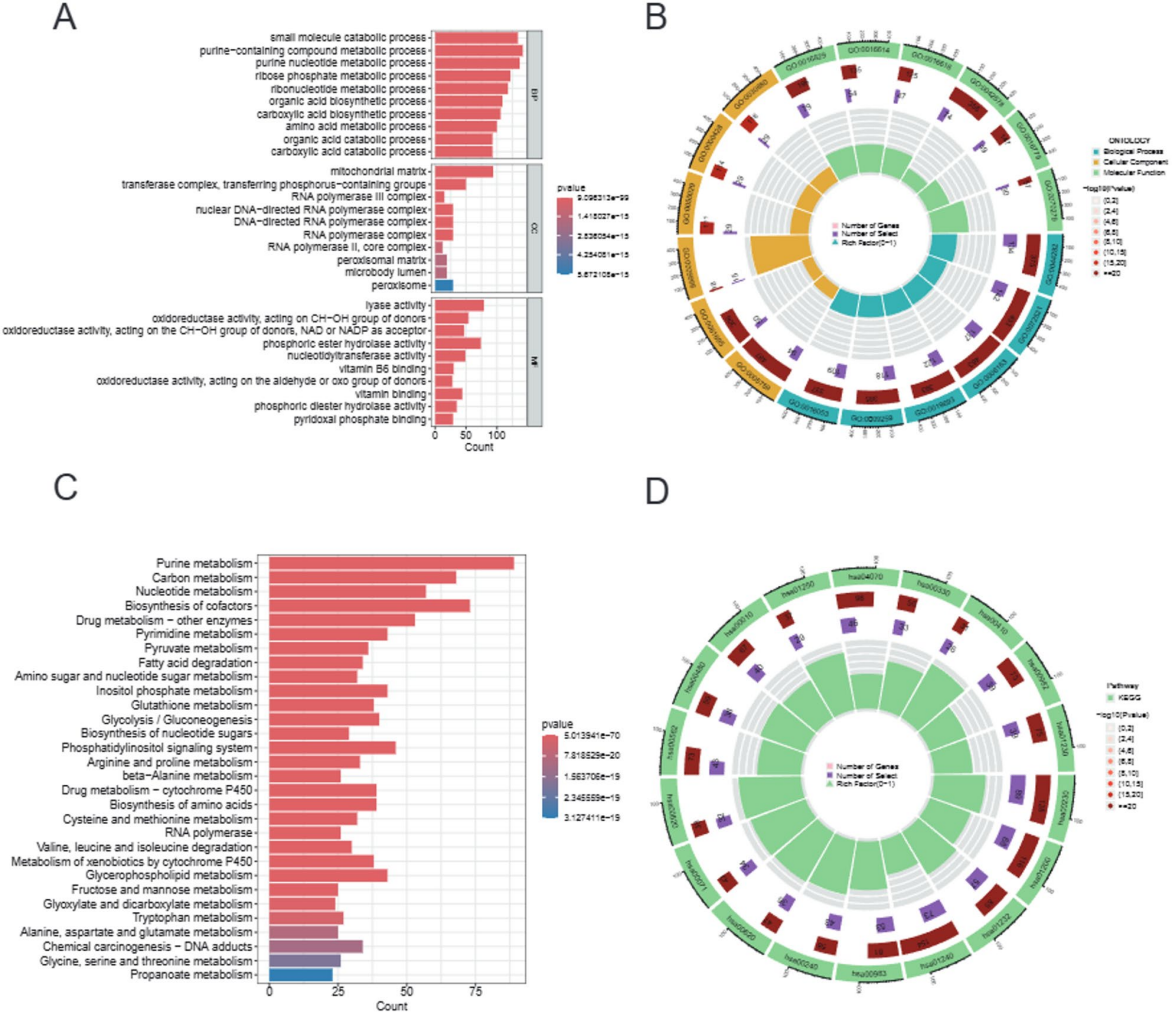
GO and KEGG Enrichment Analysis of MAGDEGs. **A**–**B** Results of GO enrichment analysis for MAGCOXs: The bar chart illustrates significant enrichment of genes across the three main GO categories—Biological process (BP), cellular component (CC), and molecular function (MF). The circular diagram visualizes the relationship between key genes and enriched functions, with color and size indicating the significance and degree of enrichment, respectively. **C–D** Results of KEGG pathway enrichment analysis for MAGCOXs: The bar chart demonstrates the key metabolic pathways involved in the diverse processes of pancreatic cancer, including purine metabolism, cofactor biosynthesis, and drug metabolism. The circular diagram showing the gene-pathway association, highlighting the significance of purine metabolism and inositol phosphate metabolism

Gene Ontology (GO) and Kyoto Encyclopedia of Genes and Genomes (KEGG) enrichment analyses were performed on the differentially expressed MAGCOXs to comprehensively identify functional modules associated with the pathogenesis of pancreatic cancer. Within the biological process (BP) category, GO analysis revealed (Figs. 2A–B) that differential genes were significantly enriched in small molecule catabolic processes, purine-containing compound metabolic processes, and nucleotide biosynthetic pathways (FDR < 1e-10), indicating extensive metabolic reprogramming within the tumor microenvironment (TME). In the molecular function (MF) category (indicated by a green box in Fig. 2B), GO analysis revealed enrichment for oxidoreductase and phosphoric diester hydrolase activities, further validating dysregulation in energy metabolism. The KEGG pathway analysis (Figs. 2C–D) revealed simultaneous activation of core metabolic pathways, including: ① Purine metabolism (75 genes enriched, *p* = 5.01 × 10⁻⁷), which is a central pathway in nucleotide biosynthesis essential for nucleic acid synthesis [22]; ② Biosynthesis of cofactors and drug metabolism—cytochrome P450, indicating that cancer cells sustain their proliferative pressure by enhancing detoxification capacity; ③ Inositol phosphate metabolism (*p* = 2.18 × 10⁻¹⁵), indicating potential abnormal activation of survival pathways such as PI3K-AKT-mTOR [23].

### Consensus clustering of PAAD subtypes based on MAGCOXs

**Fig. 3:**
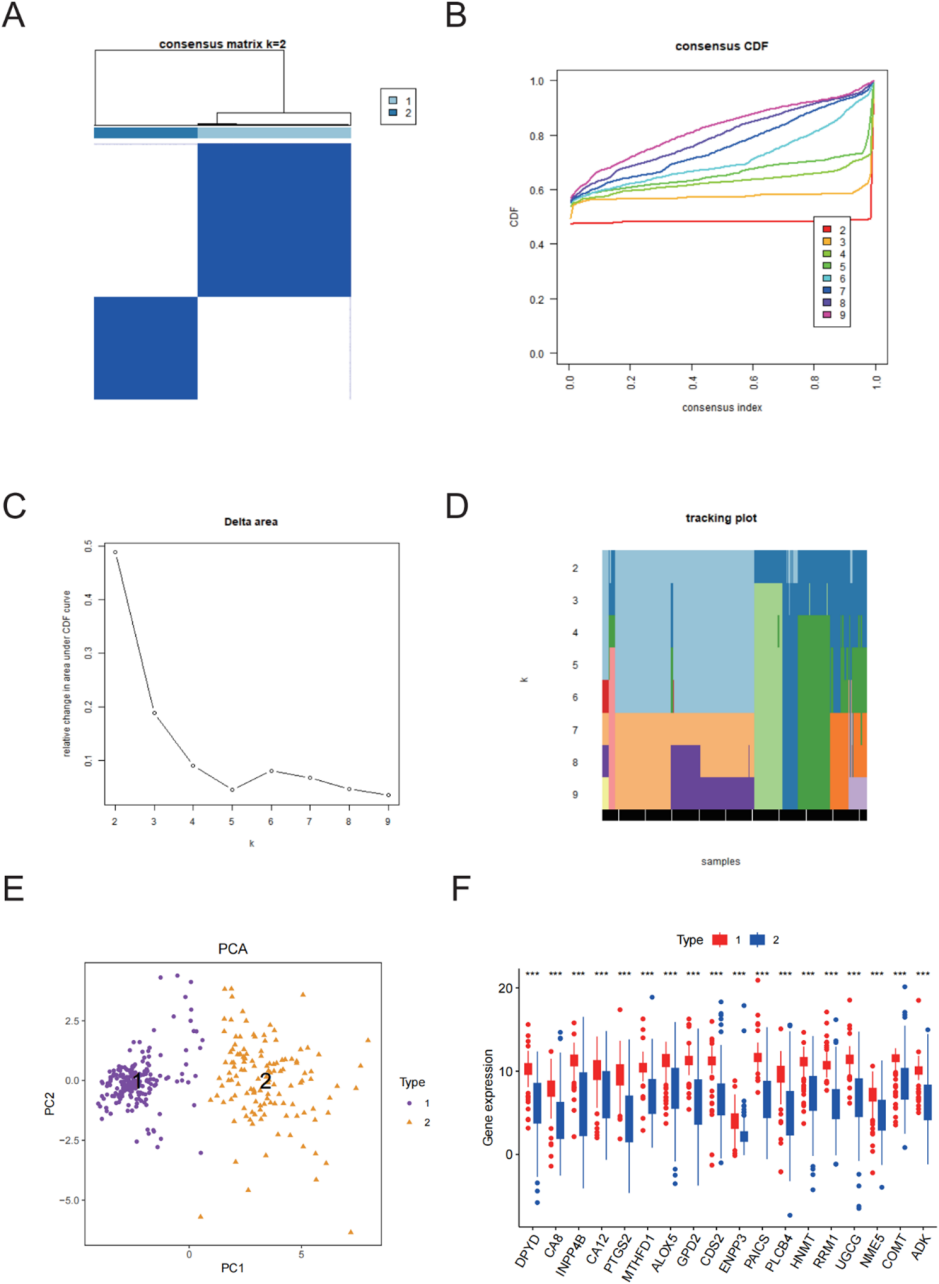
Consensus Clustering Analysis for the TCGA-PAAD and GSE57495, GSE28735. **A** A plot of the consensus clustering results for PAAD. **B**–**D** Consensus clustering analysis: cumulative distribution function (CDF) curve (**B**), Delta area plot (**C**), and sample tracking plot (**D**). **E** Principal Component Analysis (PCA) plot indicating the significant spatial distribution differences between the two PAAD subtypes. **F** Differential expression analyses for the MAGCOX-related prognostic genes between the two PAAD subtypes. *** indicates P < 0.001, ** indicates P < 0.01, and * indicates P < 0.05

To investigate the contribution of MAGCOXs to the molecular heterogeneity of pancreatic cancer, consensus clustering was performed to identify potential molecular subtypes. Analysis of the consensus matrix revealed that when the number of clusters was set to k = 2, patients were delineated into two stable subtypes, indicating significant molecular differences between the subtypes (Fig. 3A)Further assessment of clustering stability was performed using the CDF curve and Delta area analysis. The results demonstrated that clustering consistency achieved its peak at k = 2, where the Delta value also peaked, validating that k = 2 as optimal number of clusters (Fig. 3B-C).

The sample tracking plot showed that, at k = 2, sample distribution was the most stable, with minimal sample switching between subtypes. This observation further supports the robustness and consistency of the clustering results (Fig. 3D).

Notably, PCA (Fig. 3E) indicated a clear separation of the two subtypes in reduced-dimensional space, reflecting their distinct metabolic profiles. Results of differential expression analysis indicated that the 19 MAGCOXs showed marked differential expression between the two subtypes, suggesting that they are important biomarkers for classifying and prognosing pancreatic cancer (Fig. 3F).

### Construction of a prognostic model based on MAGCOXs and survival analysis

**Fig. 4:**
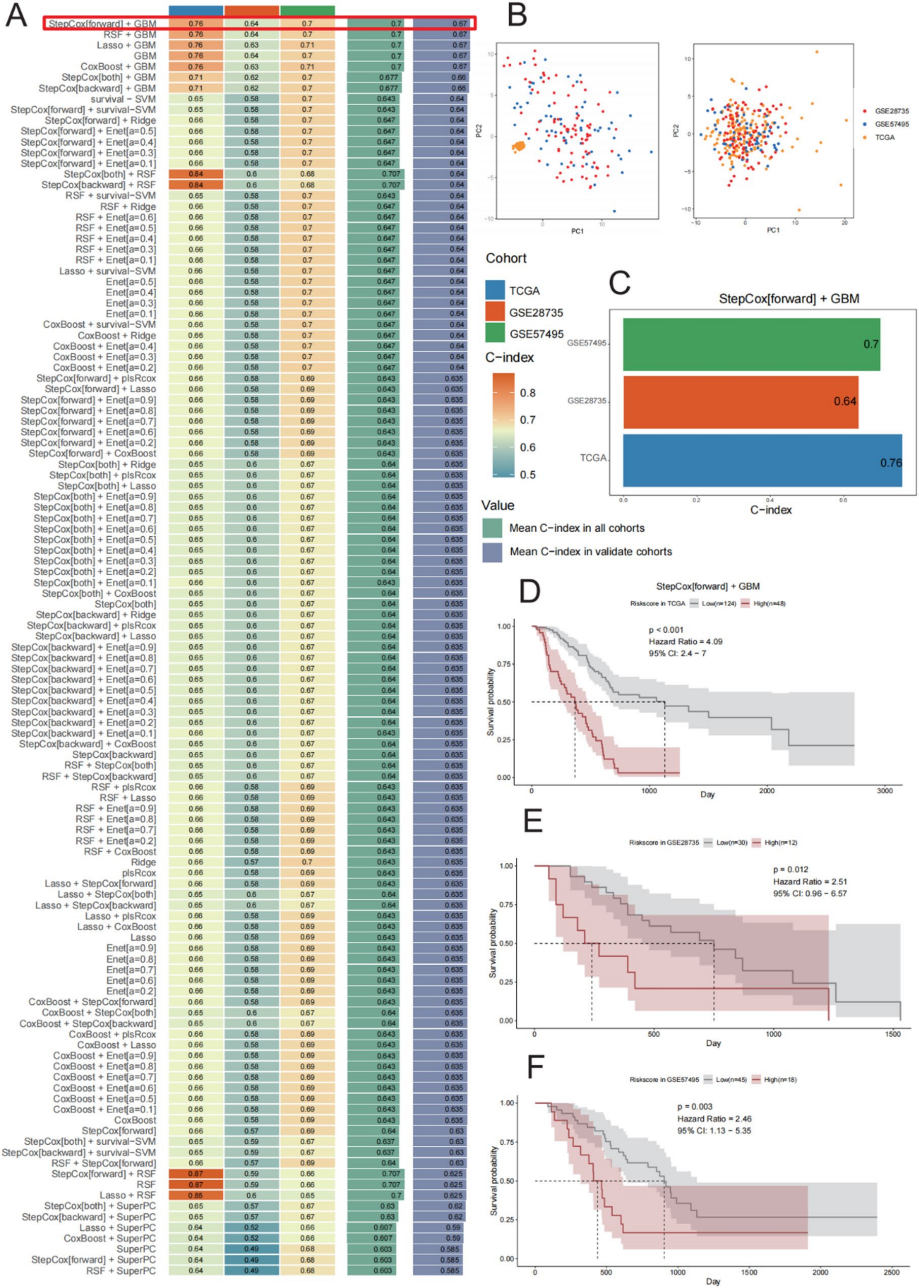
Construction of Prognostic Models in TCGA-PAAD, GSE57495, and GSE28735 Cohorts. **A** Heatmap of concordance index (C-index) values among different models based on 101 machine learning algorithm combinations in the TCGA, GSE28735, and GSE57495 cohorts. **B** Batch-corrected PCA plot illustrating the distribution of TCGA-PAAD, GSE57495, and GSE28735 samples in expression profile space. **C** Performance of the StepCox[forward] + GBM model across different cohorts, indicated by specific C-index values. **D**–**F** Kaplan–Meier survival curve analyses of risk scores from the StepCox[forward] + GBM model across different cohorts: (**D**) TCGA cohort, (**E**) GSE28735 cohort, and (**F**) GSE57495 cohort. The shaded areas indicate 95% confidence intervals

Multiple independent cohorts were analyzed to facilitate the development of a robust and reliable prognostic model for pancreatic cancer. Among the various model combinations employed in this study, the StepCox[forward] + GBM model consistently demonstrated superior performance across all cohorts. In the TCGA training cohort, the model achieved a C-index of 0.76, while in the GSE28735 and GSE57495 validation cohorts, the C-indices were 0.64 and 0.70, respectively—significantly outperforming traditional algorithms such as LASSO and Ridge. This model demonstrated strong predictive stability and generalizability across diverse datasets [24] (Fig. 4A and C).

To further evaluate the transferability of the model between datasets, batch effect removal was conducted, followed by PCA on the TCGA, GSE28735, and GSE57495 datasets. The expression profiles showed some overlap in PCA space, while still preserving distinct characteristics of each cohort, thereby validating a solid basis for data integration and comparative analysis (Fig. 4B).

Based on model-derived risk scores, patients were stratified into high- and low-risk groups, with the median score as the threshold value. Kaplan–Meier survival curves analysis revealed that the high-risk patients exhibited significantly poor survival outcomes in the TCGA cohort (HR = 4.09, 95% CI: 2.4–7.0, *p* < 0.001; Fig. 4D). Similarly, consistent patterns were observed in the GSE28735 (HR = 2.51, *p* = 0.012) and GSE57495 (HR = 2.46, *p* = 0.003) datasets (Figs. 4E–F). These results indicate that the model can reliably distinguish patients across different risk levels, underscoring its potential utility in prognostic evaluation and clinical risk stratification.

### Comprehensive validation of model predictive performance

**Fig. 5:**
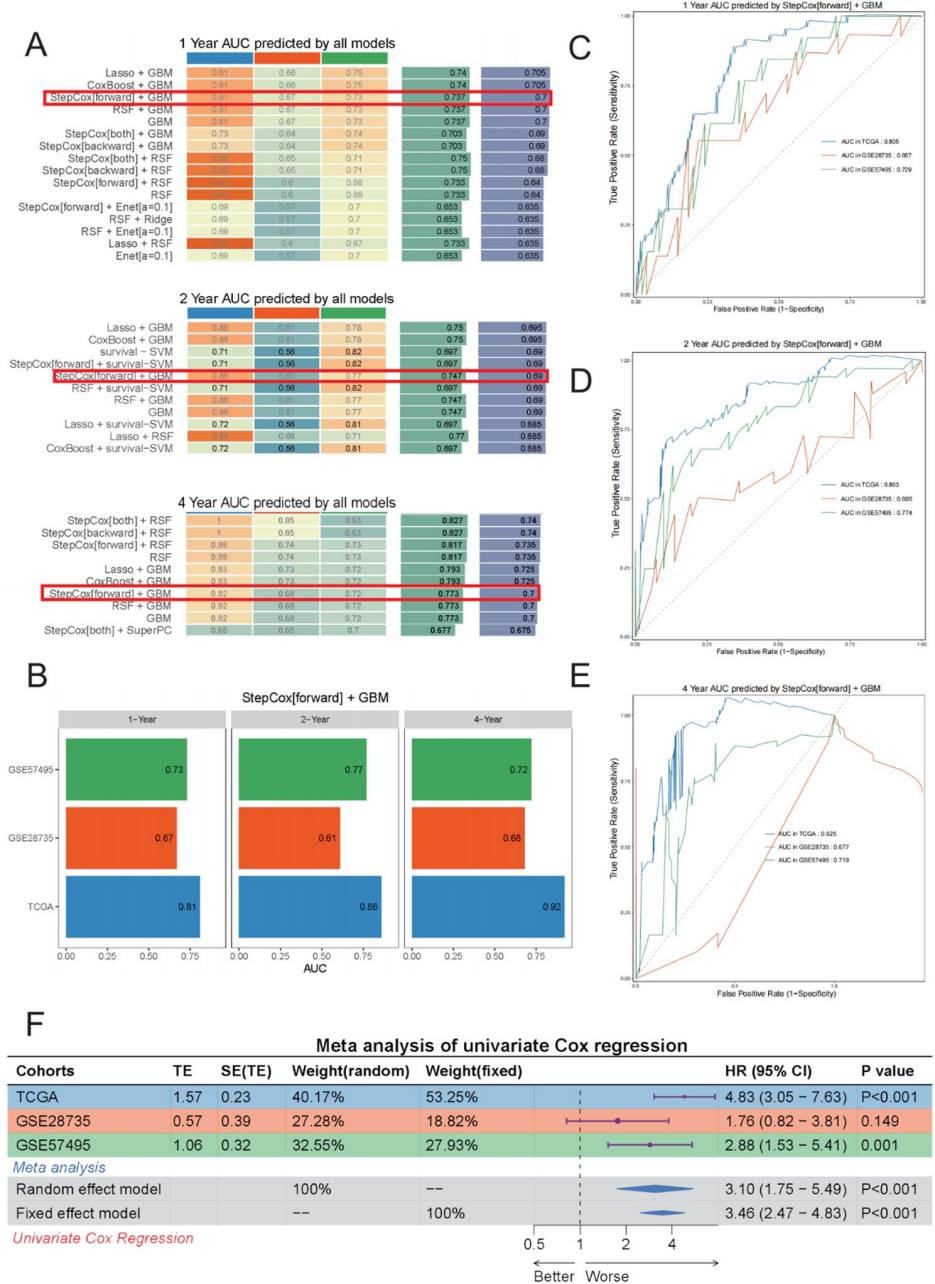
Analysis of the Predictive Performance and Statistical Validation of the Pancreatic Cancer Prognostic Model. **A** Heatmap comparing the AUC values of various machine learning models for predicting 1-, 2-, and 4-year survival rates. The red box indicates that the StepCox[forward] + GBM model had the best performance. **B** Comparison of AUC values for the StepCox[forward] + GBM model at 1-, 2-, and 4-year across the TCGA, GSE28735, and GSE57495 cohorts. C–E. Time-dependent ROC curves of the StepCox[forward] + GBM model predicting 1-year (**C**), 2-year (**D**), and 4-year (**E**) survival among different cohorts. **F** Results of univariate Cox regression meta-analysis of the risk scores of the StepCox[forward] + GBM model reveal a significant association between risk score and survival outcomes in pancreatic cancer across the three independent datasets. *** indicates p < 0.001, ** indicates p < 0.01, * indicates < 0.05

Multidimensional model assessments and meta-analyses were used to further validate and evaluate the predictive performance of the StepCox[forward] + GBM prognostic model. Time-dependent ROC curve analyses were used to compare the predictive performance of the model for 1-, 2-, and 4-year survival across different cohorts. The StepCox[forward] + GBM model demonstrated excellent AUC values across all time points, with even a sustained strong performance at the 4-year window (Figs. 5A–B). In the TCGA cohort, the AUCs for 1-, 2-, and 4-year survival predictions were 0.805, 0.863, and 0.925, respectively. Furthermore, the model maintained stable and superior predictive performance in the GSE28735 and GSE57495 validation datasets compared to other models (Figs. 5C–E).

To comprehensively explore the association between model-derived risk scores and patient prognosis, a univariate Cox regression meta-analysis was conducted by integrating the results from the TCGA, GSE28735, and GSE57495 datasets. The meta-analysis confirmed that risk score derived from the StepCox[forward] + GBM model significantly associated with poor prognostic outcomes for patients with pancreatic cancer. The random-effects model yielded an HR of 3.10 (95% CI: 1.75–5.49, *p* < 0.001), while the fixed-effects model yielded an HR of 3.46 (95% CI: 2.47–4.83, *p* < 0.001). Notably, the association between risk score and adverse survival outcomes was significantly strong in the TCGA cohort (HR = 4.83, 95% CI: 3.05–7.63, *p* < 0.001) (Fig. 5F).

These findings indicate that the StepCox[forward] + GBM model developed in this study possesses high predictive accuracy and exhibits robust generalizability. The risk score of the model consistently reflects differences in clinical outcomes among patients, highlighting its potential utility in the prognostic analysis.

### Comparison of the StepCox[forward] + GBM model with previously published gene expression-based prognostic models

**Fig. 6:**
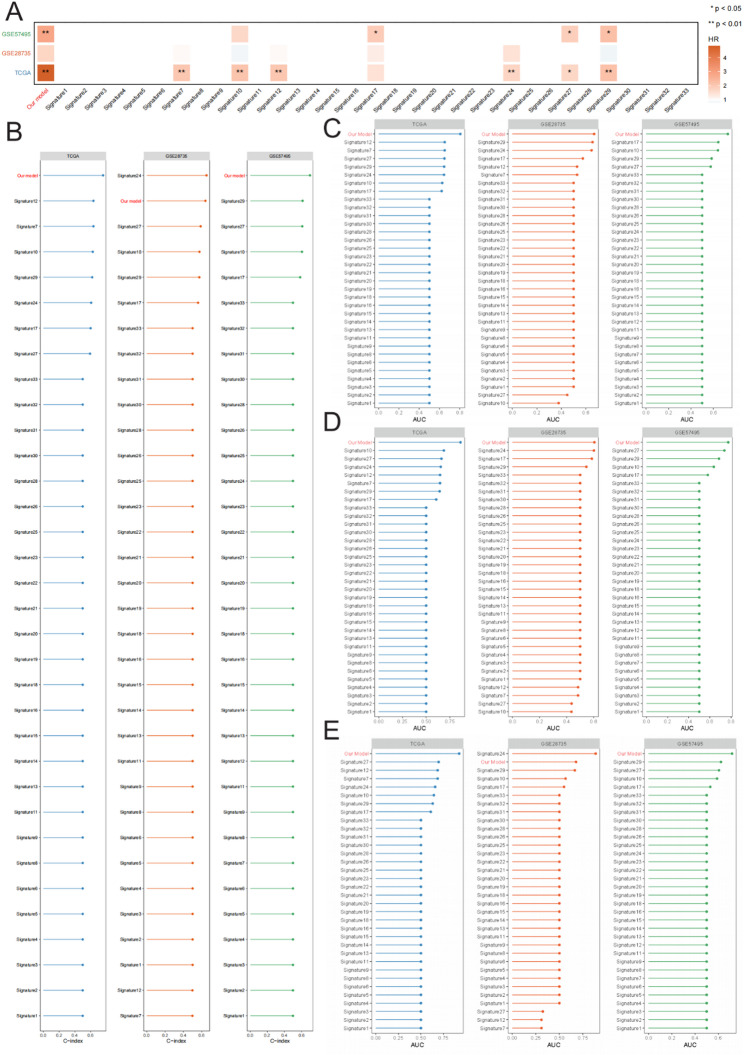
Comparison of the StepCox[forward] + GBM Model with Previously Established Prognostic Models for Pancreatic Cancer. **A** Univariate Cox regression analysis comparing the hazard ratios (HRs) of 33 published prognostic models with the StepCox[forward] + GBM model across three independent cohorts. **B** Comparison of concordance index (C-index) values between the StepCox[forward] + GBM model and the 33 previously published models in the TCGA, GSE28735, and GSE57495 cohorts. **C**–**E** Comparison of time-dependent ROC curve AUC values between the StepCox[forward] + GBM model and 33 previously published models for 1-year (**C**), 2-year (**D**), and 4-year (**E**) survival prediction

A systematic comparison was conducted to further validate the superiority of the StepCox[forward] + GBM prognostic model against 33 previously published prognostic models for pancreatic cancer across different datasets. Using univariate Cox regression analysis, the HRs of each model were evaluated. Across the three datasets, the StepCox[forward] + GBM model exhibited the strongest and most statistically significant positive correlation with poor survival outcomes, with HRs exceeding those of the majority of existing models. This indicates that the model exhibits superior prognostic discrimination between patients with good and poor outcomes (Fig. 6A).

When comparing C-indices, the StepCox[forward] + GBM model consistently outperformed other methods in nearly all cohorts, with significantly higher values compared to those of the majority of existing models (Fig. 6B), thereby demonstrating strong robustness and generalizability across independent datasets.

Furthermore, time-dependent ROC curve analyses comparing 1-, 2-, and 4-year survival predictions showed that the StepCox[forward] + GBM model consistently achieved top-tier AUC values at each time point. Notably, the model achieved significantly superior performance in the long-term 4-year survival prediction (Figs. 6C–E). These results affirm the predictive accuracy of the model and further support its potential for clinical application.

### Multidimensional validation of prognostic model performance

To further evaluate the clinical utility of the prognostic model, we conducted multivariate analyses to verify the independent prognostic value of the StepCox[forward] + GBM model. Subsequently, we constructed a nomogram incorporating traditional clinical parameters for individualized risk prediction.

**Fig. 7:**
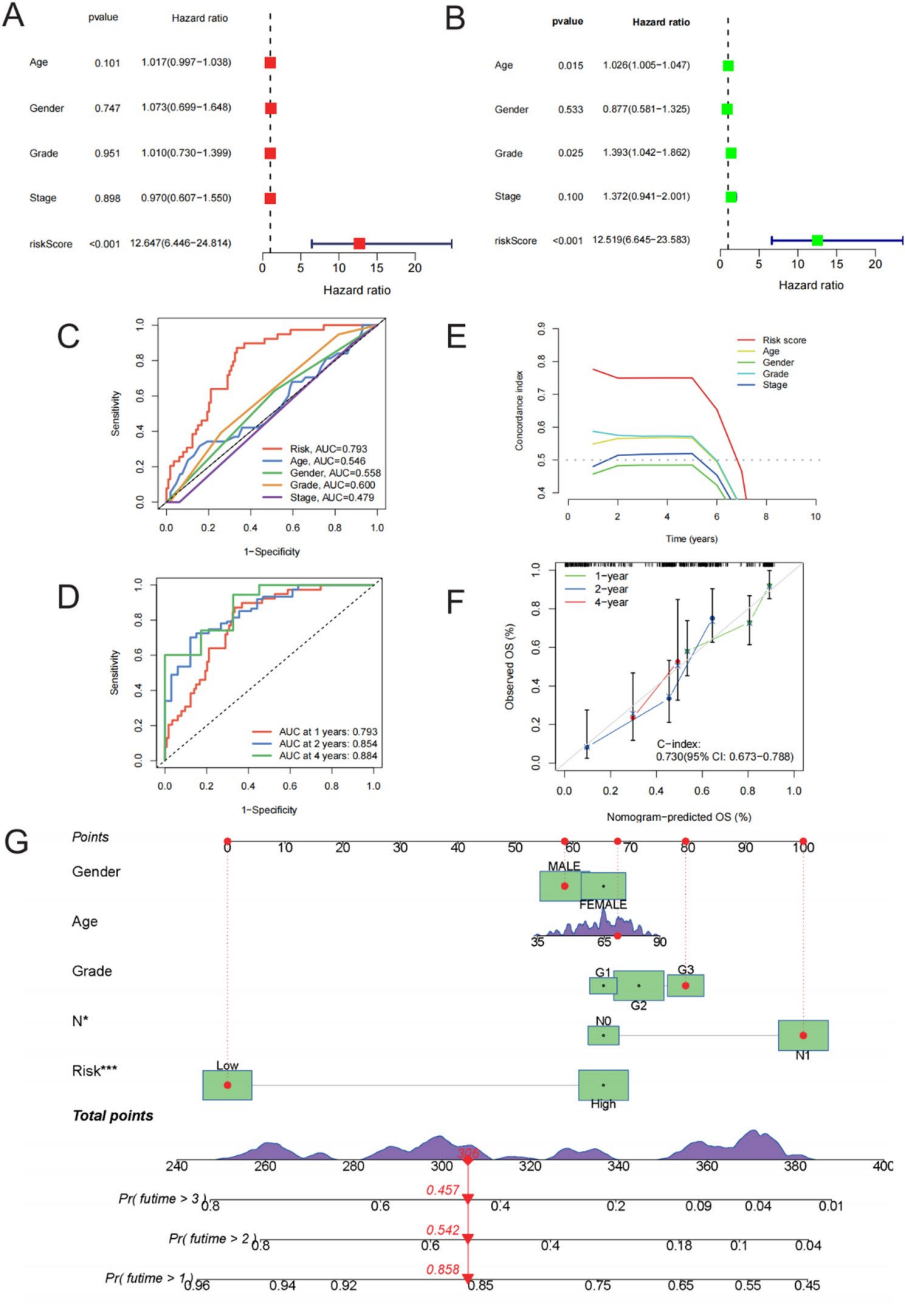
Independent Prognostic Prediction Using the Prognostic Model and Nomogram. **A**–**B** Multivariate Cox regression analyses for the training set (TCGA) and validation set (GSE), which revealed the independent prognostic role of the risk score. **C** The ROC curve comparing the prognostic accuracy of common clinical parameters and the model-derived risk scores. **D** Time-dependent ROC analysis for the risk scores in predicting the 1-, 2-, and 4-year survival. **E** Comparison of the concordance index (C-index) among various clinical parameters and the risk score. **F** Calibration curves for analyzing the accuracy of the nomogram in predicting 1-, 2-, and 4-year survival. **G** The personalized nomogram prediction tool constructed from the risk score and clinical variables to predict the survival probability among pancreatic cancer patients. In Cox regression analyses, the risk score was modeled as a continuous variable, and hazard ratios are reported per 1-unit increase in risk score

Univariate and multivariate Cox regression analyses were conducted on the model-derived risk score and conventional clinical variables (including age, gender, stage, and grade) in both the TCGA training set and external GSE validation cohorts. In both cohorts, the risk score emerged as an independent prognostic factor for poor prognostic outcomes in pancreatic cancer patients, with significantly higher HRs compared to those of other clinical variables. In the validation cohorts, the risk score was modeled as a continuous variable, and the reported hazard ratios correspond to a 1-unit increase in risk score. Because the risk score spans an approximate range of ~ 1.8–2.0 units across cohorts, the per-unit hazard ratio may appear numerically large; however, it reflects the scale of the continuous predictor rather than an overestimation of effect size. The risk score remained statistically significant in multivariate analyses (Figs. 7A–B). Schoenfeld residual testing (cox.zph) did not indicate violation of the proportional hazards assumption for the risk score (*p* = 0.678).

The prognostic accuracy of the model risk score versus traditional clinical parameters was compared using ROC curve analysis. The risk score demonstrated superior predictive accuracy, with AUCs of 0.793, 0.854, and 0.884 for 1-, 2-, and 4-year survival predictions, respectively—outperforming traditional clinical indicators such as TNM stage and age (Figs. 7C–D).

Concordance index analysis further validated the superior predictive accuracy and consistency of the risk score for survival outcomes, outperforming other conventional clinical variables (Fig. 7E). Calibration curves of the nomogram demonstrated high concordance between predicted and observed survival rates at 1, 2, and 4 years (Fig. 7F), thereby validating the model to exhibit excellent calibration and clinical applicability. A comprehensive nomogram was developed by integrating the risk score with other indicators, including clinical stage, age, and gender, to quantify individualized survival probabilities. The calibration curves derived from this integrated model showed strong concordance between predicted and actual survival outcomes for 1-, 2-, and 4-year time points, underscoring the high calibration performance and practical clinical value of the nomogram (Fig. 7G).

### Tumor immune microenvironment and immune infiltration analysis

**Fig. 8:**
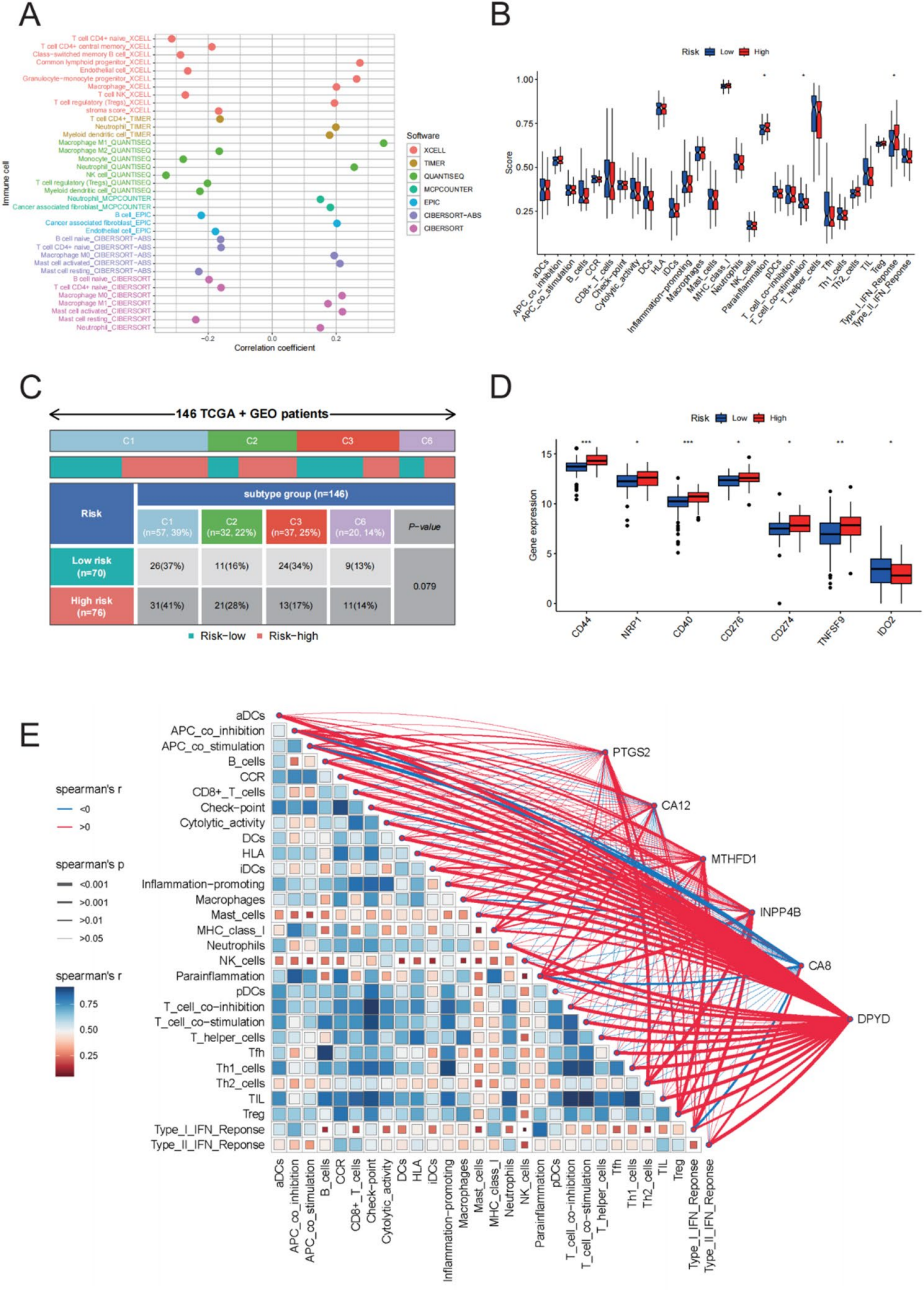
Tumor Immune Microenvironment and Immune Infiltration Analysis. **A** Heatmap showing the correlation between immune cell infiltration levels, as determined using multiple algorithms and the prognostic model risk score. **B** ssGSEA analysis displaying the enrichment results for different immune functional pathways between the high- and low-risk groups. **C** The distribution of individuals with high and low prognostic model scores for diverse immune subtypes. **D** Differential expression analysis of immune checkpoint-related genes between high- and low-risk groups. **E** Gene-immune factor interaction "butterfly plot" illustrating significant correlations between the key metabolic genes (e.g., DPYD, PTGS2) and immune infiltration status

A comprehensive analysis of immune cell infiltration was conducted to investigate the relationship between the prognostic model score and the immune microenvironment in pancreatic cancer. Multiple algorithms, including CIBERSORT-abs, EPIC, MCPCOUNTER, QUANTISEQ, XCELL, and TIMER, were employed to quantify immune cell infiltration levels. Correlation analyses using the model risk scores revealed that higher scores were positively associated with several immune cell subtypes, thereby indicating a significant immunosuppressive profile (Fig. 8A).

Further analysis using ssGSEA showed that high-risk groups exhibited upregulation of immune-related functional pathways—including inflammatory signaling and type I interferon responses—suggesting an activated or remodeled immune state (Fig. 8B). Immune subtype classification revealed that the low-risk group was predominantly enriched in the C1 immune-active phenotype, while the high-risk group was more frequently associated with the C2 immunosuppressive subtype. These findings suggest distinct immune microenvironment characteristics and differential immunotherapy response potential across risk strata (Fig. 8C).

Immune checkpoint gene analysis indicated that immunosuppressive factors such as CD44, CD40, and TNFSF9 were significantly upregulated in the high-risk group. This observation is consistent with an immunosuppressive phenotype (Fig. 8D). Additionally, a “butterfly plot” was used to illustrate the interaction network between model-derived metabolic genes and immune infiltration: PTGS2 was found to be positively correlated with regulatory T cells (Tregs) and negatively with cytotoxic cells, while DPYD showed strong positive correlations with immune checkpoint markers like PD-1 and CTLA-4—suggesting an association with an immunosuppressive signaling context and potential relevance to immunotherapy response (Fig. 8E).

### IPS, TIDE, and TMB Analyses

To evaluate potential immunotherapy responsiveness across risk strata, IPS (TCIA), TMB, and TIDE were compared between high- and low-risk groups. Subsequently, three key immune indicators were analyzed to evaluate its predictive potential for immunotherapy response.

**Fig. 9:**
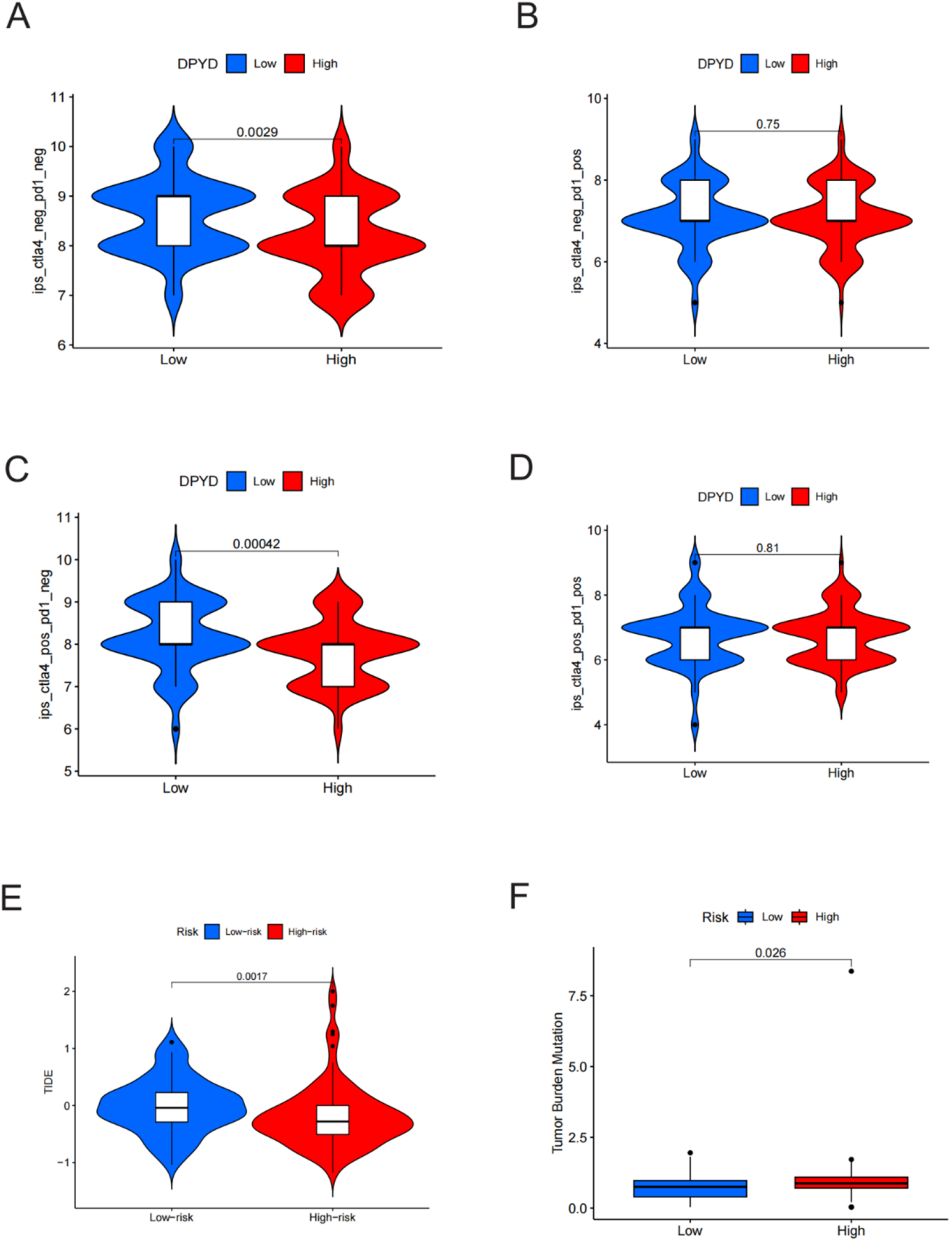
IPS, TIDE, and TMB Analyses. **A**–**D** Comparisons of the Immunophenoscore (IPS) associated with the model gene DPYD in the context of different immunotherapies: (**A**) CTLA4(−)/PD1(−), (**B**) CTLA4(−)/PD1(+), (**C**) CTLA4(+)/PD1(−), and (**D**) CTLA4(+)/PD1(+). **E**–**F** Comparison of TMB scores (**E**) and TIDE scores (**F**) between high- and low-risk groups using the prognostic model

The TCIA database was used to retrieve IPS data for TCGA-PAAD. Under varying immunotherapy contexts, IPS comparisons between high- and low-risk groups revealed that in conditions such as CTLA4(−)/PD1(−) and CTLA4(+)/PD1(−), the high-risk group had significantly lower IPS scores compared to the low-risk group, indicating weaker immune activation and poorer potential responsiveness to immunotherapy (Figs. 9A–D).

Further analysis showed that TMB was significantly lower in the high-risk group compared to the low-risk group (*p* < 0.005, Fig. 9E), which may be associated with reduced immunogenicity in a correlative manner. Additionally, the high-risk group exhibited significantly elevated TIDE scores (*p* < 0.05, Fig. 9F), suggesting a higher predicted propensity for immune dysfunction and exclusion. Collectively, the associations between the model-derived risk score and multiple immunotherapy-related indicators (IPS/TMB/TIDE) are consistent with an immunosuppressive TME context and suggest potential relevance of DPYD to immunotherapy responsiveness; however, these findings are observational and require further validation to establish causality.

### Drug sensitivity characteristics and differential analysis

**Fig. 10:**
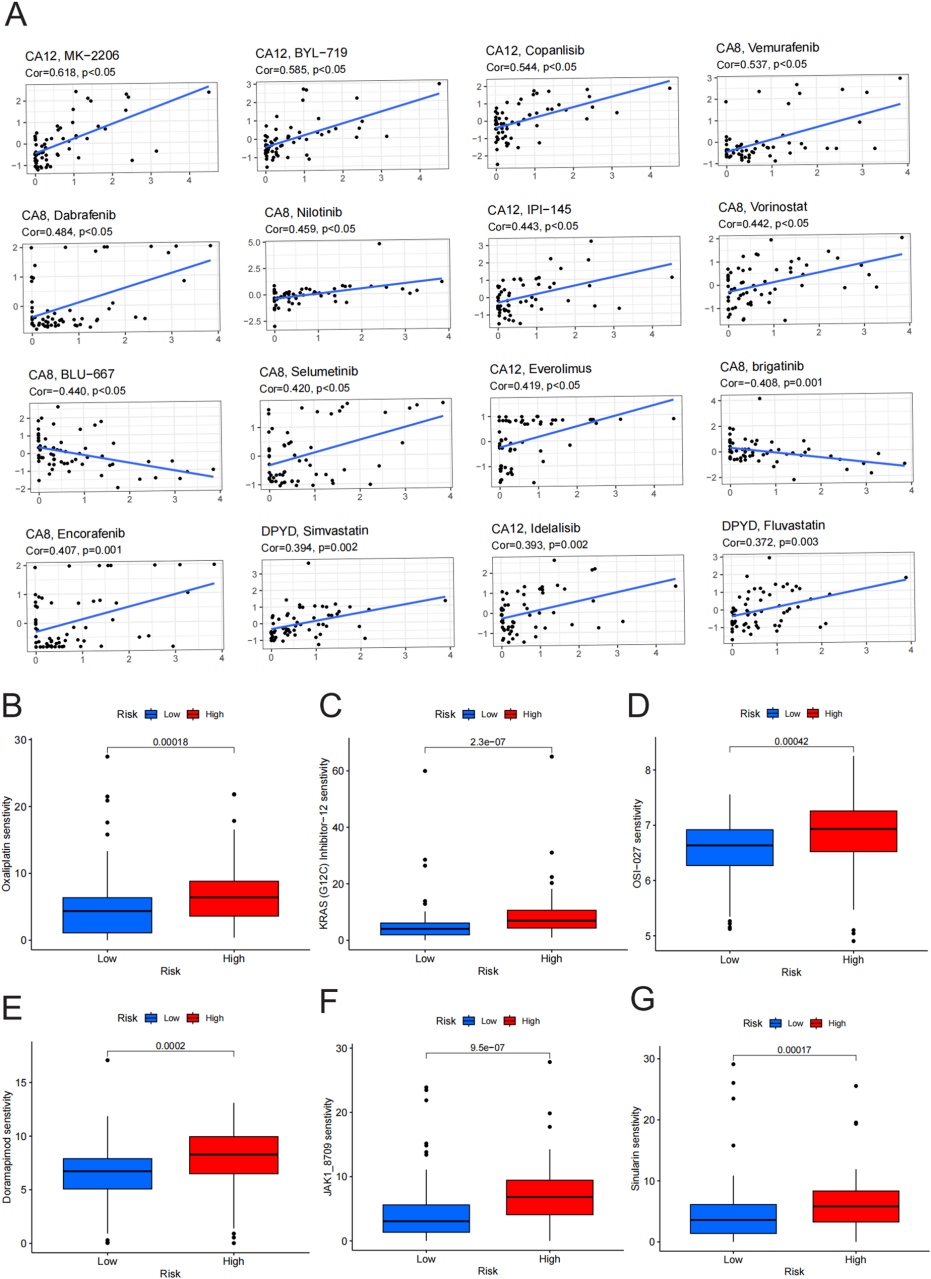
Association of Drug Sensitivity with Model Gene Scores and Expression Profiles. **A** CellMiner database analysis demonstrating the top 16 drugs that were significantly associated with DPYD expression, with simvastatin and fluvastatin showing the strongest associations. **B**–**G** Comparison of differences in the predicted sensitivity to six commonly used anticancer drugs—dabrafenib (**B**), niraparib (**C**), duvelisib (**D**), vorinostat (**E**), pralatrexate (**F**), and selumetinib (**G**)—between high- and low-risk groups

We analyzed the correlation between the expression of model genes and the sensitivity to FDA-approved or clinically used antitumor drugs to explore the potential pharmacological relevance of the key model gene DPYD in pancreatic cancer therapy. Our analysis revealed varying drug sensitivities associated with DPYD expression, with most of the association being positive. Notably, DPYD expression was moderately positively correlated with sensitivity to the HMG-CoA reductase inhibitors, simvastatin and fluvastatin (*r* = 0.394, *p* = 0.002; *r* = 0.372, *p* = 0.003), suggesting an association between DPYD expression and statin sensitivity in cell line–derived pharmacogenomic data [25] (Fig. 10A). Furthermore, DPYD demonstrated significantly stronger associations with drug response compared to other model genes, such as CA12 and CA8, thereby prioritizing DPYD as a candidate for further therapeutic exploration.

Notably, these drug–gene associations are exploratory because CellMiner is based on pan-cancer cell line models, and pRRophetic provides in silico sensitivity estimates; therefore, PAAD-specific experimental validation and/or clinical evidence will be required before any therapeutic implications can be inferred.

Further evaluation of differences in drug sensitivity using the risk score of the model revealed that six chemotherapeutic agents—dabrafenib, niraparib, duvelisib, vorinostat, pralatrexate, and selumetinib—were significantly different between high- and low-risk groups. Specifically, high-risk patients were more sensitive to vorinostat and niraparib, with higher predicted sensitivity to vorinostat and niraparib in the high-risk group (Figs. 10B–G); however, these estimates are computational and should be interpreted as hypothesis-generating until validated in PAAD-relevant experimental systems.

### Single-cell RNA-seq and trajectory analysis

To further explore the cellular origin and functional status of key metabolic genes in pancreatic cancer, particularly focusing on the expression patterns of DPYD within the TME, scRNA-seq data was systematically integrated into the model to investigate the cellular composition and trajectory evolution in PAAD and adjacent non-tumorous tissues (ADJ).

**Fig. 11:**
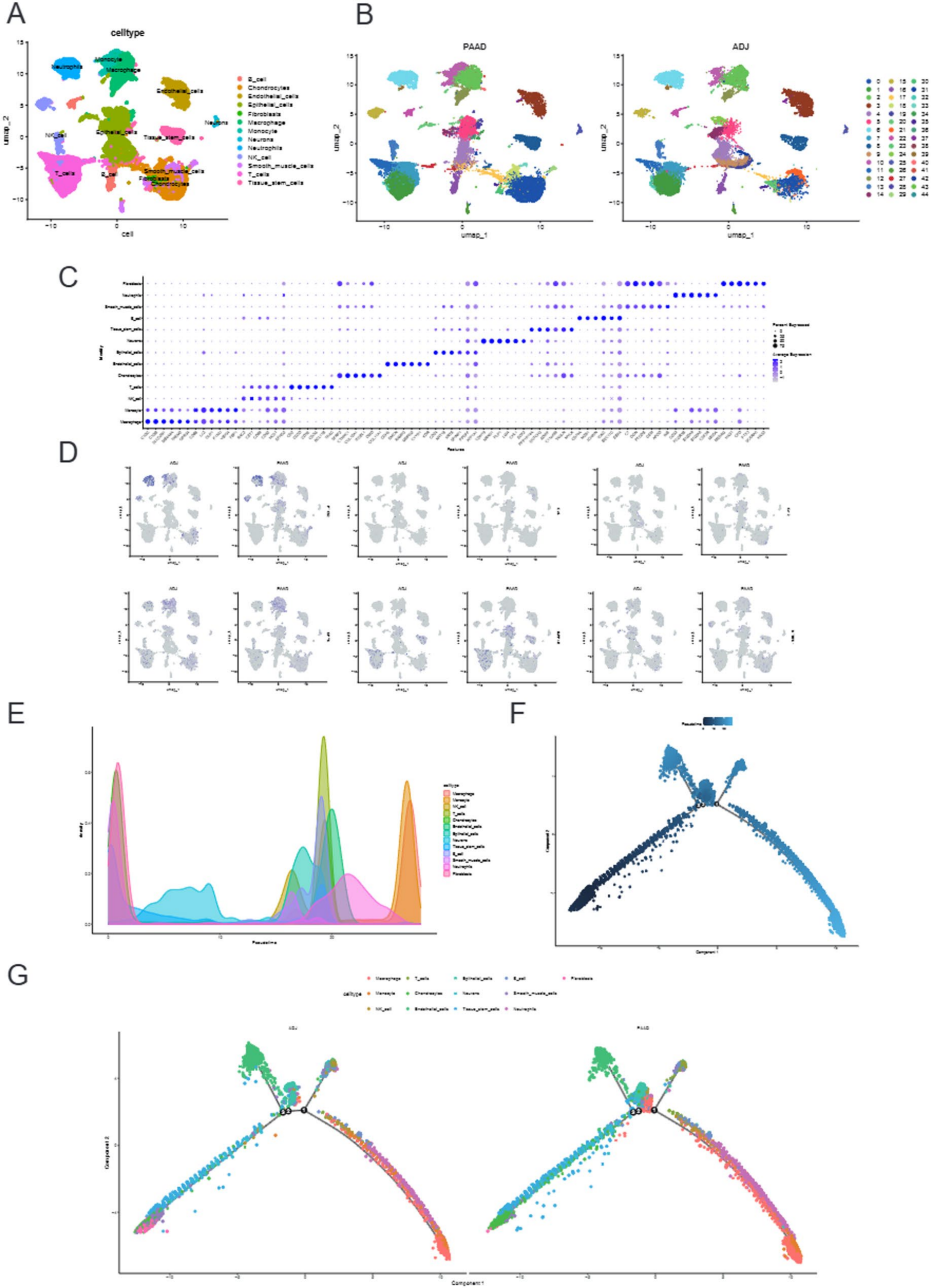
Single-Cell Transcriptomic Analysis Reveals Cellular Heterogeneity and Trajectory Dynamics in PAAD and Adjacent Tissues. **A** UMAP plot showing the clustering of various cell types, such as macrophages, monocytes, NK cells, T cells, B cells, chondrocytes, epithelial cells, neurons, endothelial cells, fibroblasts, smooth muscle cells, neutrophils, and tissue stem cells. **B** UMAP plot comparing the cell distribution between PAAD and adjacent (ADJ) tissues. **C** The distribution of typical marker genes across different cell subpopulations. **D** Feature plots illustrating the expression distribution of key model genes (including DPYD, PTGS2, INPP4B, and MTHFD1) across different cell types in the UMAP space. **E** The density plot showing the distribution of different cell types and their pseudotime trajectory. **F** Pseudotime trajectory plot derived from Monocle2 analysis. **G** Cell trajectories colored by cell type and grouped by tissue origin, illustrating dynamic changes in specific cell lineages between PAAD and ADJ tissues

Analysis using UMAP clustering identified multiple cell subpopulations, including macrophages, monocytes, T cells, B cells, NK cells, fibroblasts, and epithelial cells. Coloring based on tissue origin revealed significant differences in cellular composition between PAAD and ADJ tissues, indicative of immune and stromal remodeling in the TME (Figs. 11A–B). Furthermore, the expression patterns of canonical marker genes—such as CD3D, MS4A1, CD68, and EPCAM—were visualized to verify the accuracy of cell type annotation. The analysis indicated the occurrence of distinct and specific expression across cell types, validating the clustering results (Fig. 11C).

Analysis of the expression profiles of key metabolic genes from the model (such as DPYD, PTGS2, INPP4B, and MTHFD1) across cell types revealed that DPYD was primarily enriched in macrophages and specific T cell subsets, suggesting an association with immune-related cell states and an immunosuppressive context within the TME (Fig. 11D). These single-cell findings primarily delineate the cellular sources of DPYD expression and its association with immune-related states in the tumor microenvironment, rather than establishing a direct functional role in these immune subsets. Pseudotime trajectory analysis using Monocle illustrated dynamic developmental trends among various cell types. Notably, there was a clear transition from resting to activated states, particularly within immune cells. Coloring based on the tissue origin, the trajectory plots revealed that several immune subpopulations in PAAD tissues were significantly shifted along the pseudotime axis, indicating that their developmental fates experienced remodeling by the TME (Figs. 11E–G).

Single-cell transcriptomic analysis revealed the presence of immune and stromal heterogeneity in pancreatic cancer. Additionally, this analysis identified critical metabolic genes such as DPYD with distinct expression patterns and functional transitions, suggesting potential relevance to immune-related states and tumor progression.

### DPYD promotes tumor migration and invasion in pancreatic cancer cells

**Fig. 12:**
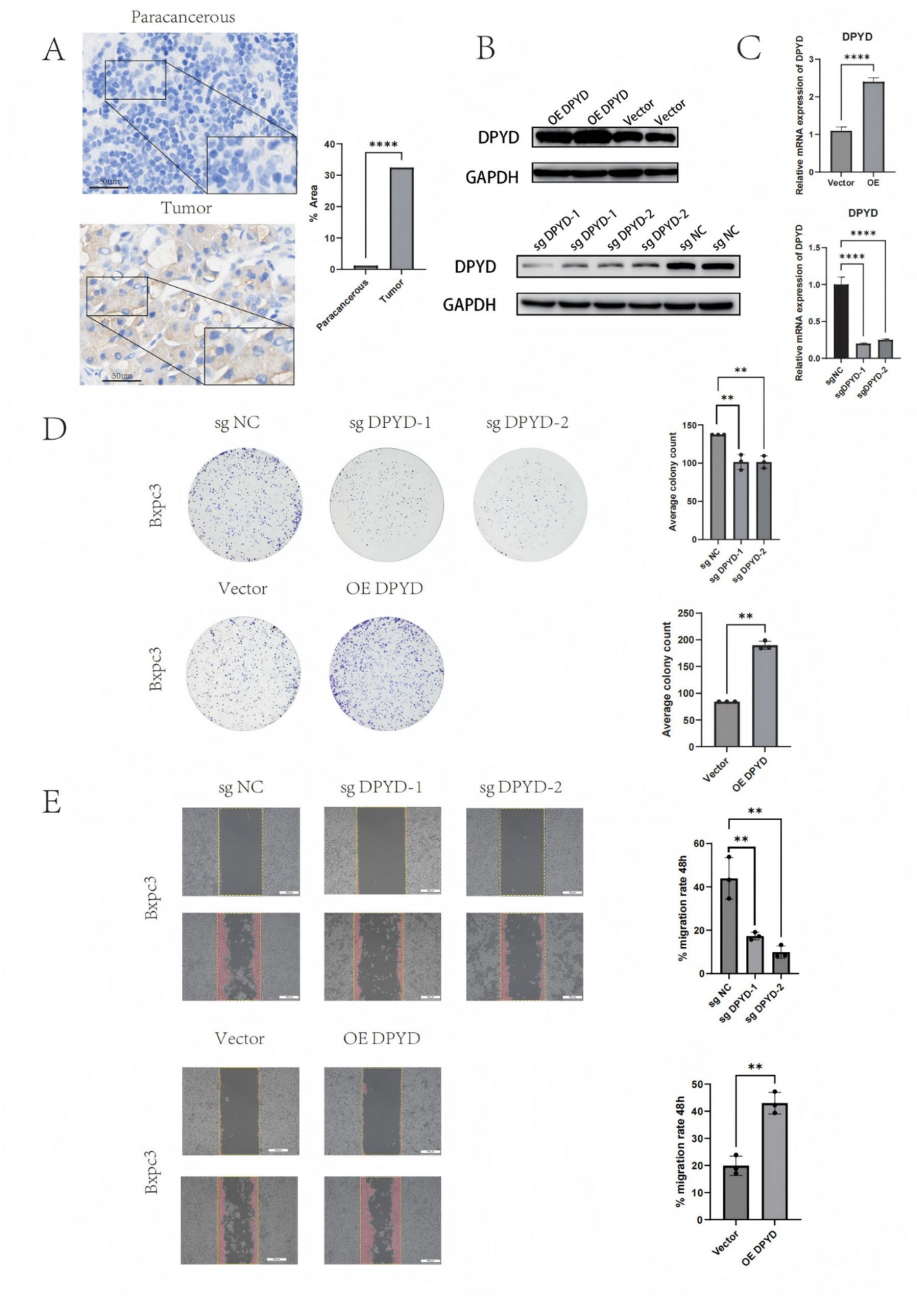
Functional Validation of DPYD in Pancreatic Cancer Cells. **A** Representative immunohistochemistry (IHC) images showing elevated expression of DPYD in pancreatic cancer tissues (Tumor) relative to their adjacent non-tumorous tissues (Paracancerous). **B**–**C** Western blot (**B**) and qRT-PCR (**C**) demonstrating the expression levels of DPYD in BxPC-3 cells with stable overexpression (OE) and knockdown (shDPYD-1/2). **D** Colony formation assays indicating that DPYD knockdown suppressed the clonogenic ability, whereas its overexpression strongly enhanced it. **E** Wound healing assays illustrating that silencing DPYD delayed the cell migration, whereas its overexpression accelerated wound closure. Data are expressed as the mean ± standard deviation. **** indicates P < 0.0001, ** indicates p < 0.01. Full-length Western blot images are provided in Supplementary materials

Given that scRNA-seq analyses highlighted prominent DPYD expression in macrophages and specific T-cell subsets, we next performed tumor cell–based functional assays to evaluate whether DPYD also exerts tumor-intrinsic effects that could contribute to malignant phenotypes in PAAD cells. To systematically validate the expression level and functional role of DPYD in pancreatic cancer, IHC analysis was employed. The results revealed significantly higher DPYD expression in tumor tissues compared to ADJ, indicating its upregulation in pancreatic cancer (Fig. 12A). Stable DPYD overexpression and knockdown (sgDPYD-1/2) BxPC-3 cell lines were generated, and successful modulation of DPYD expression was validated using qRT-PCR and Western blotting at the transcript and protein levels, respectively (Figs. 12B–C).

Functional assays revealed that the clonogenic ability of BxPC-3 cells was significantly suppressed following DPYD knockdown, whereas overexpression of DPYD significantly enhanced proliferative capacity. Wound healing assays further showed that cell migration capacity was strongly correlated with DPYD expression: silencing DPYD delayed wound closure, while its overexpression promoted cell migration, thereby enhancing wound closure (Figs. 12D–E).

Collectively, these findings suggest that DPYD is overexpressed in PAAD and is associated with enhanced proliferative and migratory phenotypes in pancreatic cancer cells, supporting DPYD as a candidate biomarker and a potentially actionable metabolic vulnerability that warrants further validation in vivo and in clinically relevant settings.

### Enrichment analysis of differential proteins after DPYD knockdown

To further investigate the molecular mechanisms regulated by DPYD, quantitative proteomic mass spectrometry analysis was performed on BxPC-3 cells with DPYD knockdown (shDPYD). Quantitative label-free proteomic analysis was conducted with three independent biological replicates per group (*n* = 3). Differentially expressed proteins were identified under the criteria of fold change ≥ 1.2 and Benjamini–Hochberg adjusted *P* < 0.05. GO, KEGG, and DO enrichment analyses were subsequently performed on the identified differentially expressed proteins (DEPs).

**Fig. 13:**
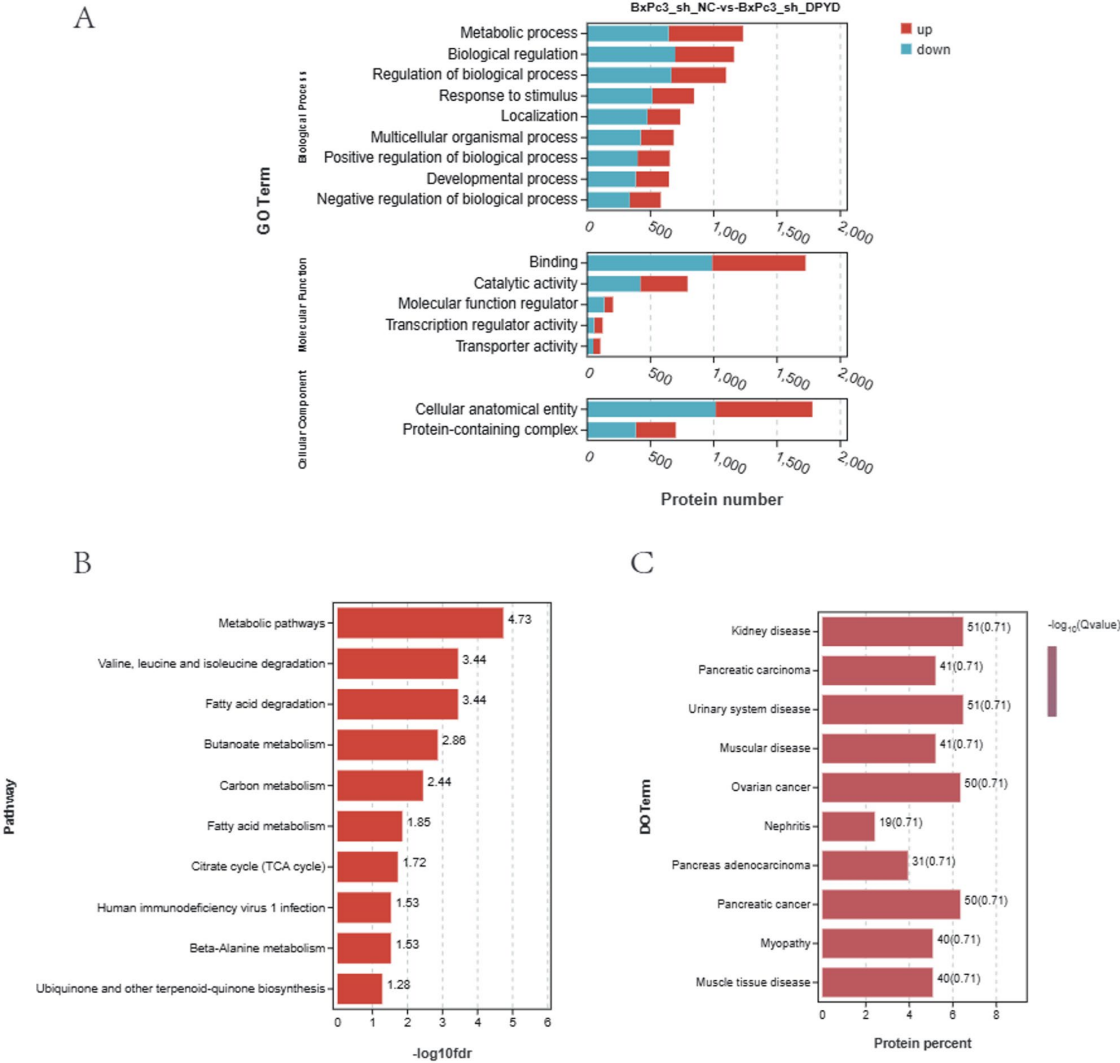
**A** GO functional enrichment analysis results showing the top enriched terms and their up- or down-regulated trends in the Biological Process (BP), Molecular Function (MF), and Cellular Component (CC) categories following DPYD knockdown (shDPYD). **B**. KEGG pathway enrichment results displaying the significantly enriched differential proteins in various metabolic pathways after DPYD knockdown (shDPYD). **C**. Disease Ontology (DO) enrichment results showing the potential associations of the differentially expressed proteins with multiple diseases, such as pancreatic cancer, muscular disorders, and kidney diseases

The GO analysis revealed that in the BP category, DPYD knockdown (shDPYD) significantly enriched genes associated with metabolic processes, biological regulation, stimulus response, developmental processes, and multicellular organismal processes. In the MF category, DEPs were primarily implicated in the binding activity, catalytic activity, transcriptional regulation, and transport functions. In the CC category, enriched terms included cellular anatomical structures and protein complexes (Fig. 13A).

The KEGG pathway enrichment analysis indicated that DPYD knockdown (shDPYD) resulted in alterations of proteins predominantly enriched in various metabolism-related pathways, including fatty acid, butanoate, carbon, and branched-chain amino acid metabolisms, as well as the TCA cycle. These findings suggest that DPYD plays multiple roles in regulating energy metabolism and biosynthesis (Fig. 13B).

Notably, disease ontology (DO) enrichment analysis revealed a significant enrichment of these DEPs in multiple disease categories, particularly in pancreatic cancer, ovarian cancer, muscular disorders, and nephritis. Notably, enrichment analysis revealed that terms such as “pancreatic cancer,” “pancreatic adenocarcinoma,” and “urinary system disease” were highly enriched, suggesting that DPYD may contribute to the pathogenesis of multiple diseases through the regulation of associated protein networks (Fig. 13D).

## Discussion

In this study, we successfully constructed a robust prognostic model for PAAD based on 19 key metabolic genes, utilizing integrated multi-omics data and advanced machine learning algorithms. The model demonstrated excellent predictive stability, discriminative ability—as indicated by high C-index and time-dependent AUCs—and generalizability across both the TCGA training cohort and independent GEO validation datasets (GSE28735, GSE57495). Notably, its performance was significantly superior to those of numerous previously published PAAD prognostic models. Most importantly, the model enabled accurate risk stratification of PAAD patients and uncovered the intricate relationship between metabolic reprogramming and the immunosuppressive tumor microenvironment. Consistent with the Results section, the high-risk group displayed an immunosuppressive immune contexture and poorer predicted immunotherapy responsiveness, supporting a potential link between metabolic heterogeneity and immune evasion in PAAD; however, these observations remain correlative and require mechanistic validation.

A key finding of this study was the identification of DPYD as a core metabolic driver in the progression of PAAD. This conclusion was supported by multiple converging lines of evidence: ① High DPYD expression was an independent risk factor for poor prognostic outcomes in patients with PAAD and exhibits a strong clinical prognostic value. ② Single-cell transcriptomic analysis revealed that DPYD was specifically enriched in macrophages and T cell subpopulations within the TME, with distinct expression trajectories between tumor and adjacent tissues, suggesting it may be associated with immune cell states and TME features. ③ Functional in vitro experiments demonstrated that DPYD is a key regulator of malignant phenotypes in PAAD cells. Notably, DPYD knockdown significantly inhibited colony formation and migration of the BxPC-3 cells, while overexpression promoted these capabilities, thereby validating its oncogenic role. Importantly, these results address complementary biological questions at different analytical levels. The single-cell analyses were designed to identify the cellular sources of DPYD expression within the tumor microenvironment and to characterize its association with immune components, whereas the in vitro assays were performed to validate the tumor-intrinsic, cell-autonomous role of DPYD in pancreatic cancer cells. Therefore, the adverse prognostic impact of DPYD likely reflects a combined contribution of tumor-intrinsic metabolic advantages and immune microenvironment remodeling. Mechanistically, proteomic analysis following the knockout of DPYD revealed significant disruption of key metabolic pathways essential for maintaining cellular homeostasis and malignancy, including fatty acid metabolism [26, 27], the TCA cycle [28], and branched-chain amino acid catabolism [29]. Downregulation of these pathways may disrupt cellular energy homeostasis and impair biosynthetic metabolic processes. Additionally, DO analysis showed that DEPs were significantly enriched in pancreatic cancer and related diseases, supporting DPYD as a candidate metabolic regulator supporting energy production and anabolic metabolism in PAAD, consistent with enhanced tumor proliferation, invasion, and adaptation to microenvironmental stress [30]. The enrichment of DPYD in immune cells suggests that its associated metabolic dysregulation may alter the availability of critical metabolites (such as acetyl-CoA, α-ketoglutarate and branched-chain keto acids), which may be linked to immune cell differentiation and function and could potentially contribute to an immunosuppressive phenotype; this putative mechanism warrants dedicated validation (e.g., co-culture or in vivo studies) [31].

Drug sensitivity analysis provided new insights into the clinical translational potential of DPYD. Analysis of the CellMiner database revealed a positive correlation between DPYD expression and sensitivity to HMG-CoA reductase inhibitors (including simvastatin and fluvastatin). However, this finding requires cautious interpretation, as the underlying biological mechanisms remain incompletely understood [32]. Nonetheless, the potential mechanisms include a DPYD-associated metabolic state that enhances statin sensitivity, or noncanonical functions of DPYD in lipid metabolism or drug metabolism. This observation still warrants further validation in clinically relevant models. Moreover, model-based drug sensitivity predictions suggested that high-risk patients might respond better to pharmacological agents like vorinostat and niraparib, suggesting that these agents may merit prioritization for future hypothesis-driven testing in PAAD-relevant systems (e.g., patient-derived organoids/PDX models) rather than indicating actionable treatment recommendations.

Despite identifying DPYD as a key regulator and establishing a metabolic regulatory network in PAAD through integration of multi-omics analysis and functional experiments, this study has several limitations that must be acknowledged. First, functional validation was limited to in vitro models. Moreover, the tumor-intrinsic functional assays were performed in a single pancreatic cancer cell line (BxPC-3), which may limit generalizability across heterogeneous PAAD models; therefore, validation in additional PAAD cell lines and patient-derived models is warranted. In vivo validation using PDX models or conditional knockout mouse models is necessary to determine the role of DPYD in tumor progression and remodeling of the immune microenvironment [33]. In addition, although DPYD was predominantly expressed in tumor-associated macrophages in scRNA-seq analyses, its functional role in macrophages was not directly tested in this study. Future work using macrophage–tumor co-culture systems and/or in vivo models (e.g., myeloid-specific perturbation) will be required to determine whether DPYD contributes to macrophage polarization and immunosuppressive remodeling. Second, while computational tools (such as IPS and TIDE) predicted poor immunotherapy responses in high-risk groups, retrospective analyses in real-world PAAD patient cohorts who received immunotherapy are necessary to validate the predictive biomarker value of this model. Importantly, the immune infiltration analyses and immunotherapy-response indicators (IPS/TIDE/TMB) are computational and observational; therefore, they do not establish causality between metabolic risk/DPYD and immune regulation. Third, further studies using metabolomics, ChIP-seq, and Co-IP/MS are required to elucidate how DPYD precisely regulates key enzymes in downstream metabolic pathways and how either its expression or activity is modulated by upstream oncogenic or hypoxic signals. A comprehensive understanding of the downstream metabolic products governed by DPYD—and their involvement in oncogenic signaling pathways and immune regulation—is essential for uncovering potential therapeutic targets in PAAD. Finally, the causal relationship between DPYD and statin sensitivity requires experimental validation to evaluate its clinical translational potential.

In addition, several methodological considerations should be acknowledged. In the present study, tumor expression profiles were derived from the TCGA cohort, whereas normal pancreatic tissues were obtained from the GTEx database. These datasets were generated using different sequencing platforms and processing pipelines, and cross-platform batch correction was not performed in the primary differential expression analysis. Therefore, systematic technical variability between datasets may partially influence tumor–normal differential expression results. Importantly, the prognostic model construction and survival analyses were conducted exclusively within the TCGA cohort and were further validated in independent GEO datasets, thereby minimizing the potential impact of cross-dataset heterogeneity on the risk signature. Second, although 101 machine learning algorithm combinations were evaluated, the TCGA cohort size is relatively limited for extensive model exploration. Despite the use of bootstrap resampling and independent external validation, the possibility of model overfitting cannot be fully excluded. Future validation in larger multi-center or prospective cohorts will be necessary to further confirm the robustness and clinical stability of the proposed prognostic signature. In addition, although a nomogram was constructed and showed good calibration, decision curve analysis was not performed; therefore, the incremental clinical net benefit of the model over standard staging systems (e.g., TNM stage) could not be quantified in the present study. Future studies with prospective/real-world cohorts and decision-analytic evaluation are warranted to determine whether the proposed signature provides added value in clinical decision-making. Third, immune infiltration analyses were based solely on transcriptome-derived computational estimation methods. Although multiple established algorithms were applied to improve robustness, the absence of experimental validation limits direct biological interpretation. Future studies integrating single-cell sequencing or immunohistochemical validation are warranted to further confirm immune landscape alterations associated with the prognostic signature. Fourth, drug sensitivity analyses were derived from in silico prediction (pRRophetic) and pan-cancer cell line correlations (CellMiner/NCI-60), which may not fully recapitulate the pharmacogenomic landscape of PAAD; therefore, these findings should be interpreted cautiously and require validation in PAAD-relevant systems (e.g., patient-derived organoids/PDX models) before clinical translation.

## Conclusion

This study presents a prognostic model integrating metabolic and immune-related features for PAAD and highlights DPYD as a key gene associated with poor prognosis. DPYD promoted tumor-associated phenotypes in vitro and showed consistent associations with immune microenvironment features in computational analyses. Together, these findings support DPYD as a promising biomarker and a candidate metabolic target that merits further mechanistic investigation and in vivo/clinical validation, particularly to clarify its potential links to immune remodeling and therapeutic response.

## Supplementary Information

Supplementary Material 1.

Supplementary Material 2.

Supplementary Material 3.

Supplementary Material 4.

Supplementary Material 5.

Supplementary Material 6.

Supplementary Material 7.

Supplementary Material 8.

Supplementary Material 9.

Supplementary Material 10.

Supplementary Material 11.

Supplementary Material 12.

Supplementary Material 13.

Supplementary Material 14.

Supplementary Material 15.

## Data Availability

No datasets were generated or analysed during the current study.

## Abbreviations

PAAD: Pancreatic ductal adenocarcinoma
TCGA: The Cancer Genome Atlas
GEO: Gene Expression Omnibus
GBM: Gradient Boosting Machine
ROC: Receiver operator characteristic
TMB: Tumor Mutational Burden
IPS: Immunophenoscore
TIDE: Tumor Immune Dysfunction and Exclusion
DPYD: Dihydropyrimidine Dehydrogenase
WB: Western blot
qRT-PCR: quantitative Real-Time PCR
IHC: Immunohistochemistry
TCA: Tricarboxylic Acid
DEGs: Differentially Expressed Genes
FC: Fold-change
FDR: False Discovery Rate
LASSO: Least Absolute Shrinkage and Selection Operator
CIBERSORT-ABS: CIBERSORT with Absolute Score
EPIC: Estimating the Proportions of Immune and Cancer cells
MCPcounter: Microenvironment Cell Populations counter
TIMER: Tumor Immune Estimation Resource
ssGSEA: single-sample Gene Set Enrichment Analysis
NCI-60: National Cancer Institute 60 human tumor cell lines
IC50: Half Maximal Inhibitory Concentration
PDX: Patient-Derived Xenograft
ChIP-seq: Chromatin Immunoprecipitation sequencing
Co-IP/MS: Co-immunoprecipitation / Mass Spectrometry
BCA: Bicinchoninic Acid Assay
RIPA: Radio-Immunoprecipitation Assay buffer
SDS-PAGE: Sodium Dodecyl Sulfate Polyacrylamide Gel Electrophoresis
PVDF: Polyvinylidene Fluoride membrane
DAB: Diaminobenzidine
PBS: Phosphate-Buffered Saline
DMEM: Dulbecco’s Modified Eagle Medium
UMAP: Uniform Manifold Approximation and Projection
AUC: Area Under Curve
FDA: Food and Drug Administration
PI3K-AKT-mTOR: Phosphoinositide 3-Kinase / Protein Kinase B / Mechanistic Target of Rapamycin
HMG-CoA: 3-Hydroxy-3-Methylglutaryl-Coenzyme A
ADJ: Adjacent non-tumorous tissue
TME: Tumor microenvironment
