# Supplementary material for: Integrated analysis of metabolic gene features and the immune microenvironment: identification of DPYD-mediated prognostic model and therapeutic targets in pancreatic cancer
Source: BMC Gastroenterol. 2026 Mar 14;26:245. doi: 10.1186/s12876-026-04726-4 (PMC13101390; doi:10.1186/s12876-026-04726-4)
Supplement: Supplementary file 13 — Supplementary Material 13. [file 12876_2026_4726_MOESM13_ESM.pdf]

Figure 12A

T1

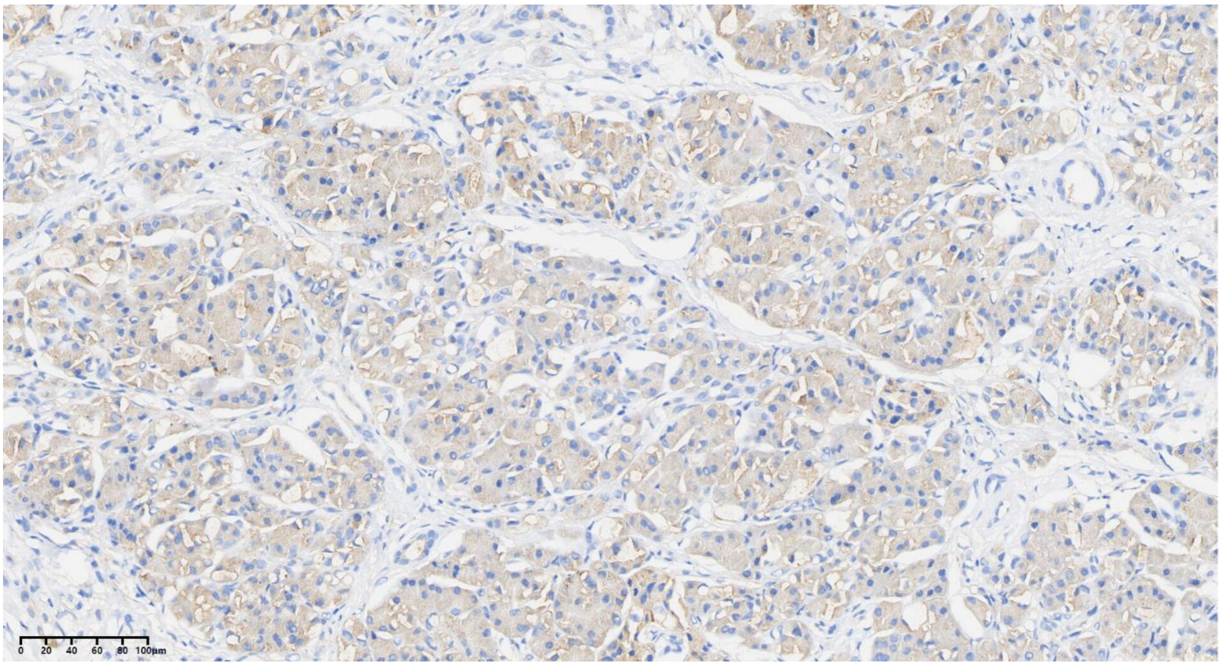

P1

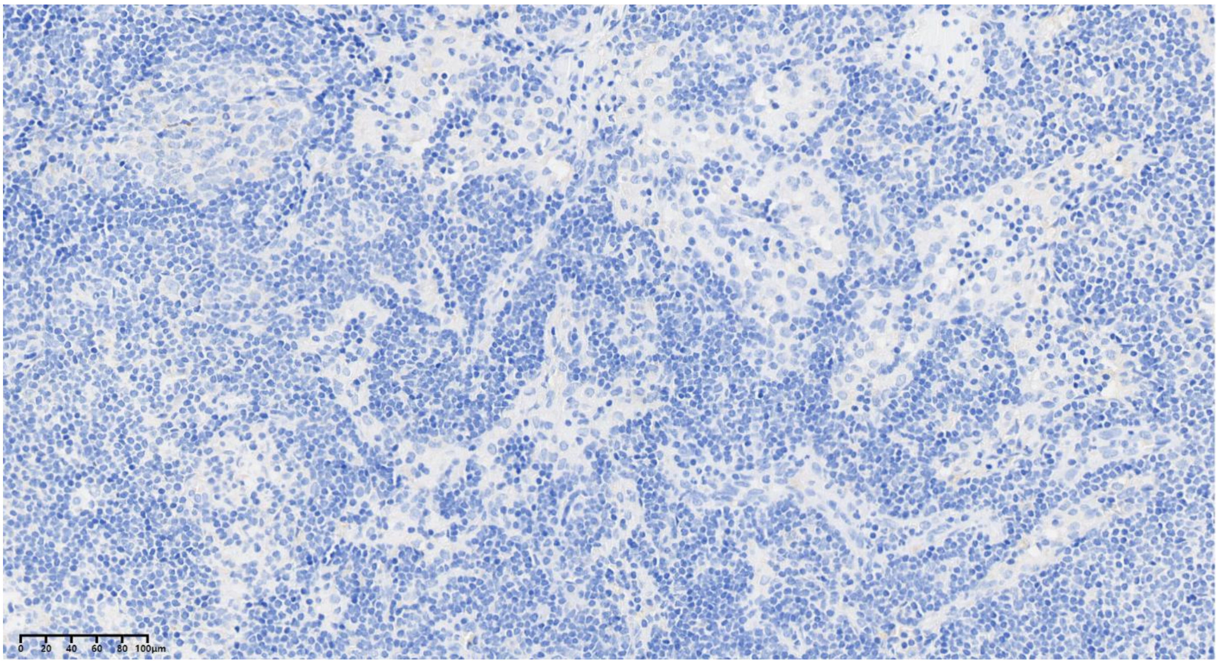

T2

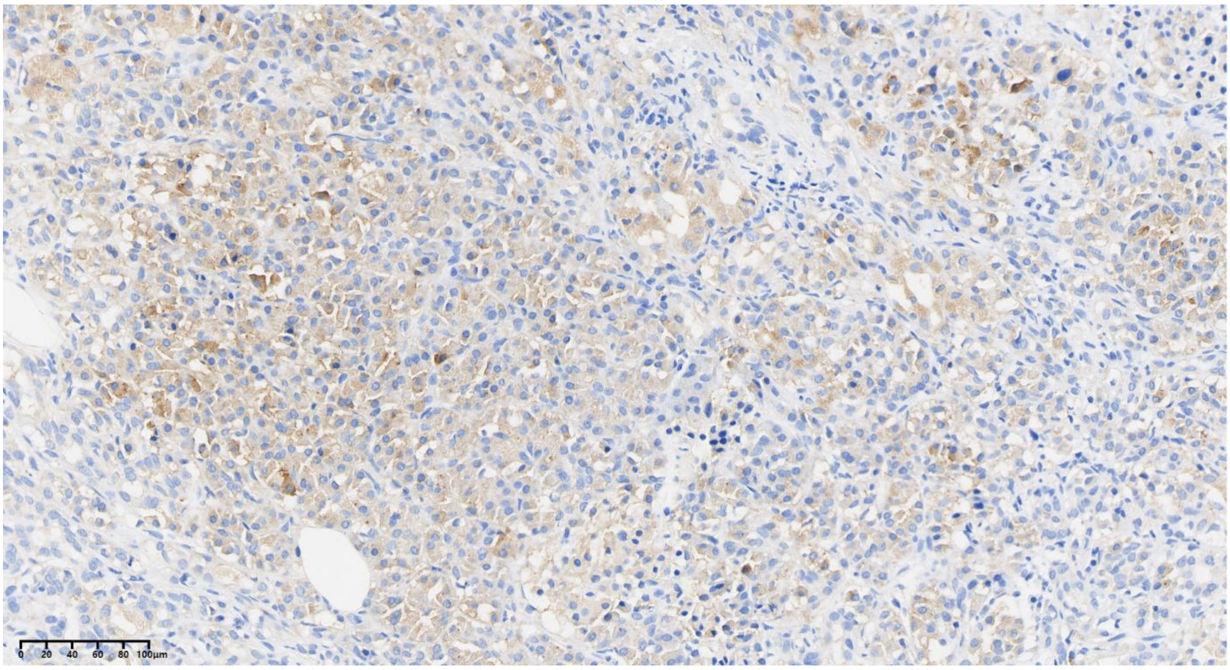

P2

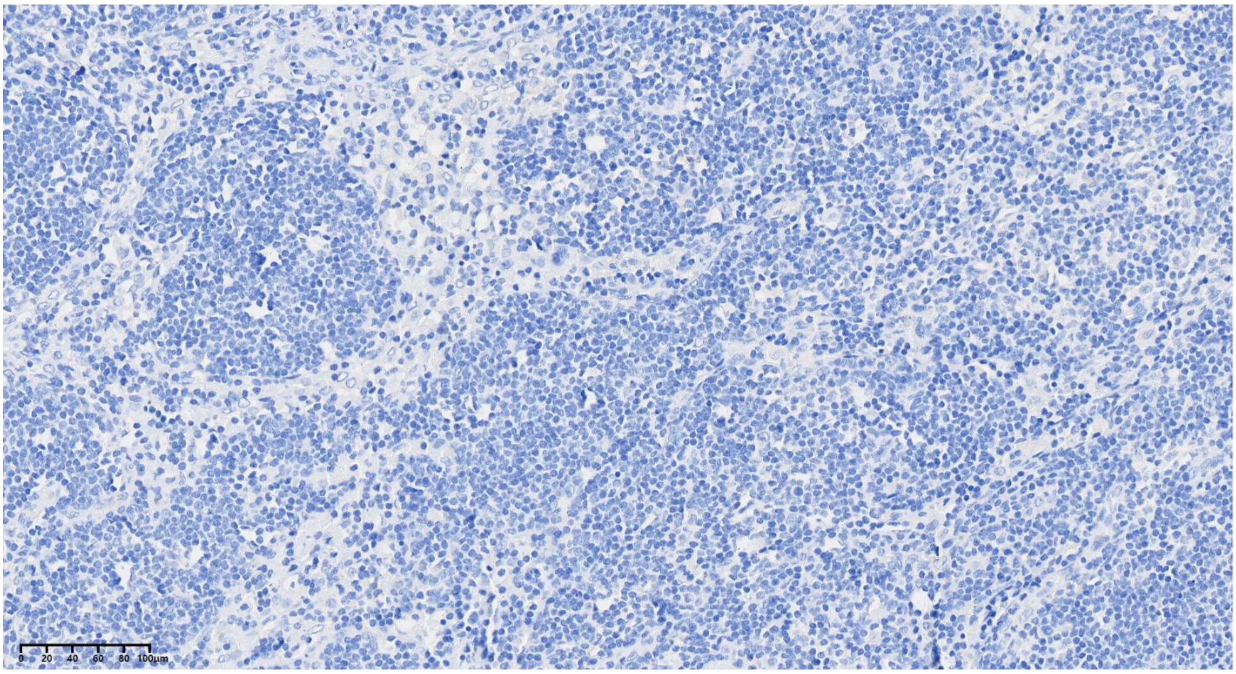

T3

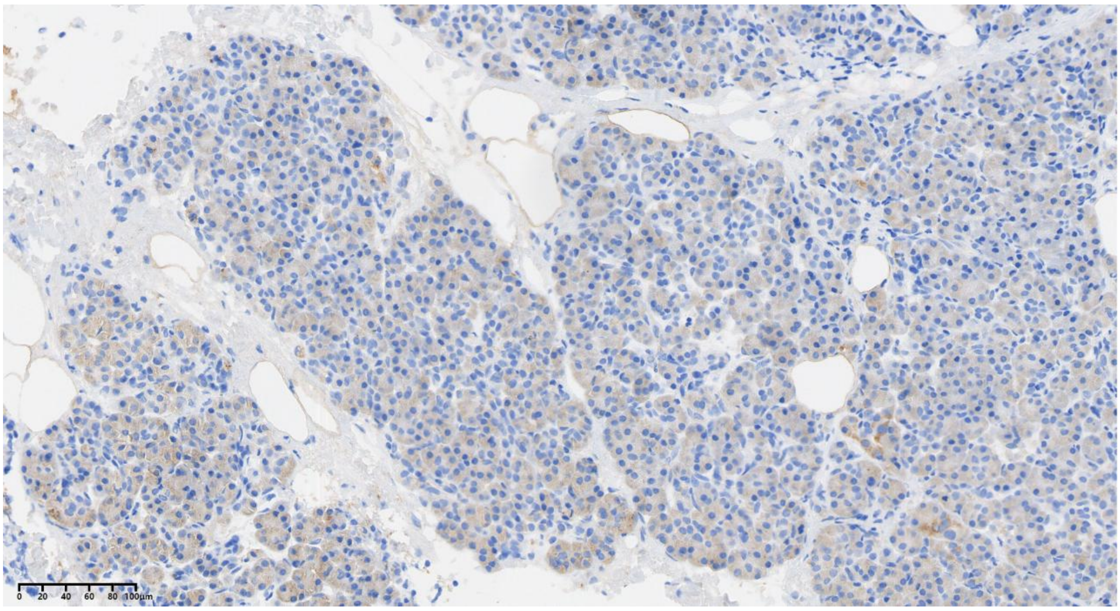
0 20 40 60 80 100µm

P3

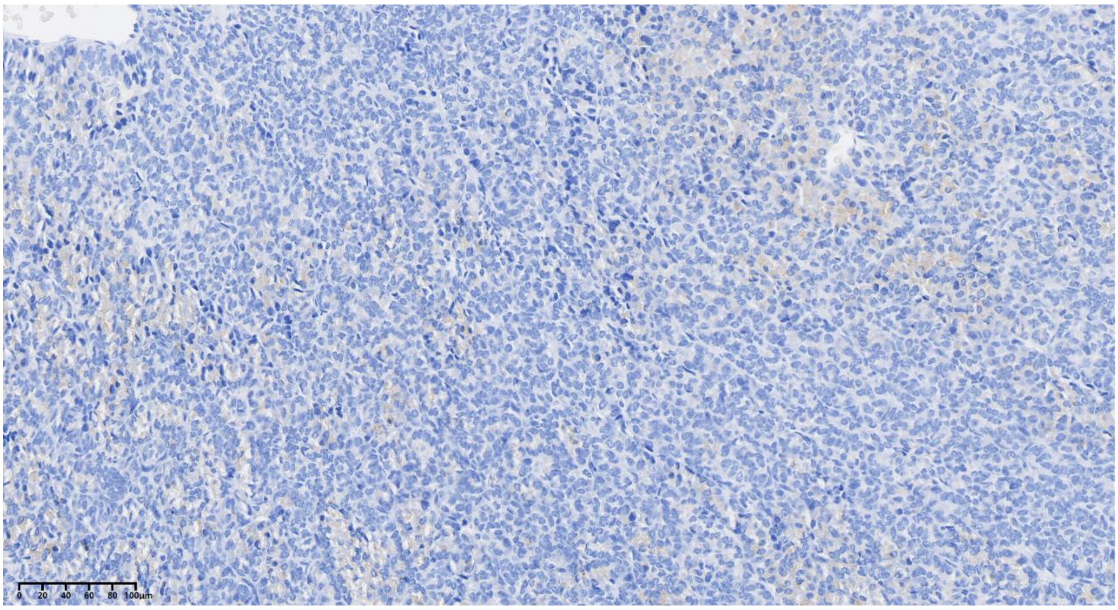
0 20 40 60 80 100µm

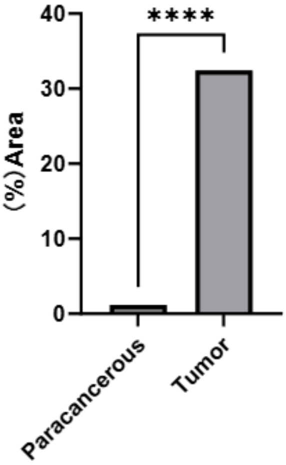
****
40
30
20
10
0
(%) Area
Paracancerous
Tumor

Figure 12B
OE-DPYD1.2

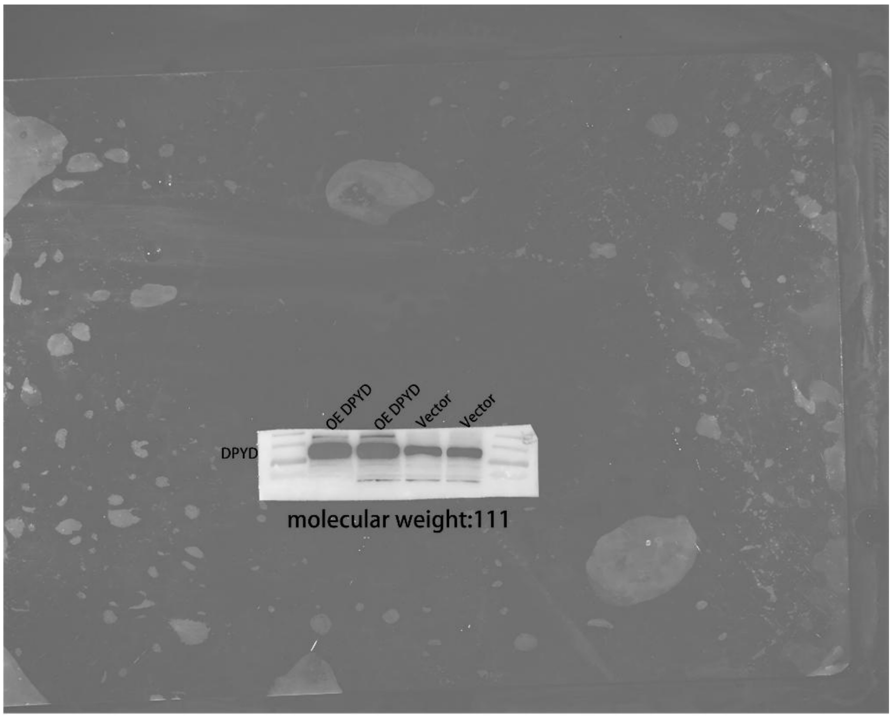

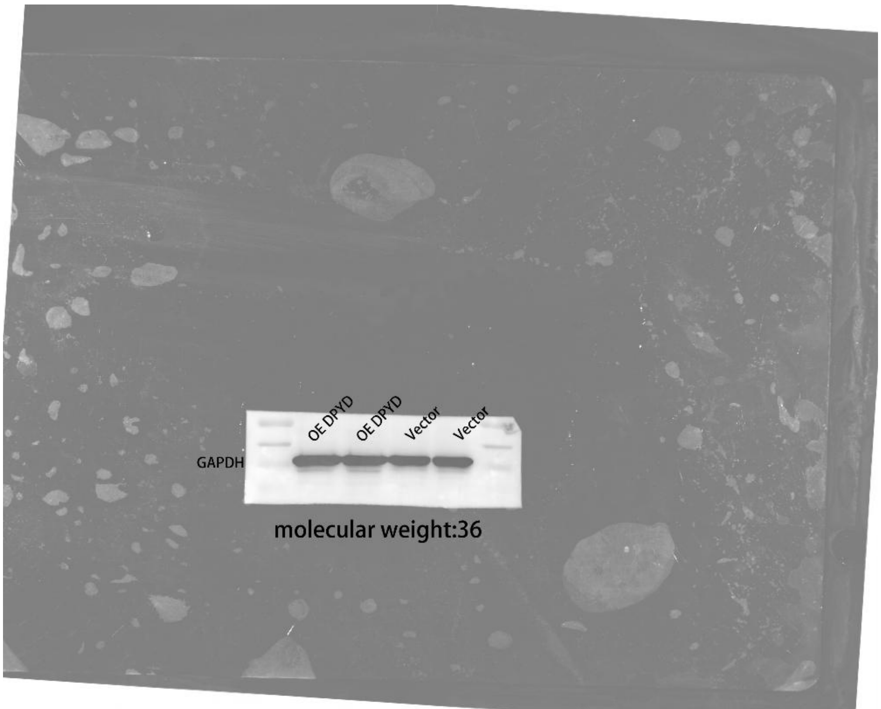

OE-DPYD1.3

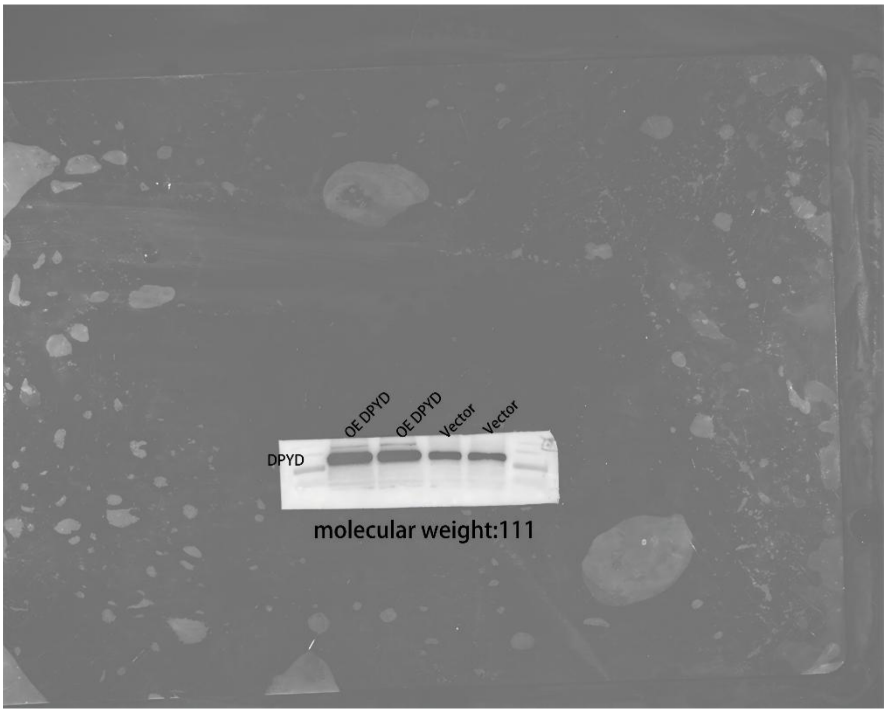
OE DPYD
OE DPYD
Vector
Vector
DPYD
molecular weight:111

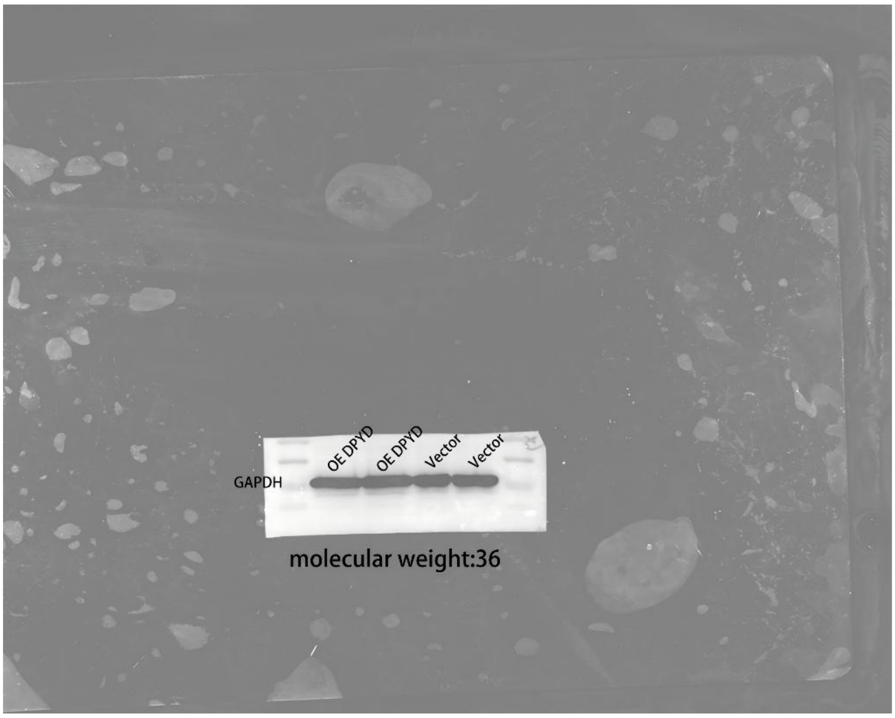
OE DPYD
OE DPYD
Vector
Vector
GAPDH
molecular weight:36

sg DPYD1-1

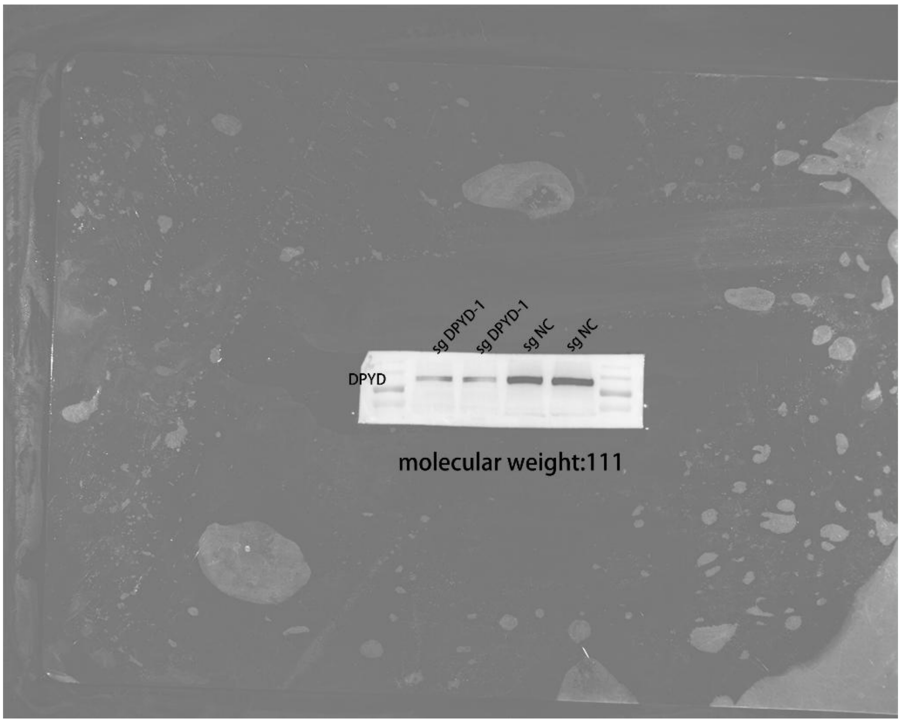
sg DPYD-1
sg DPYD-1
sg NC
sg NC
DPYD
molecular weight:111

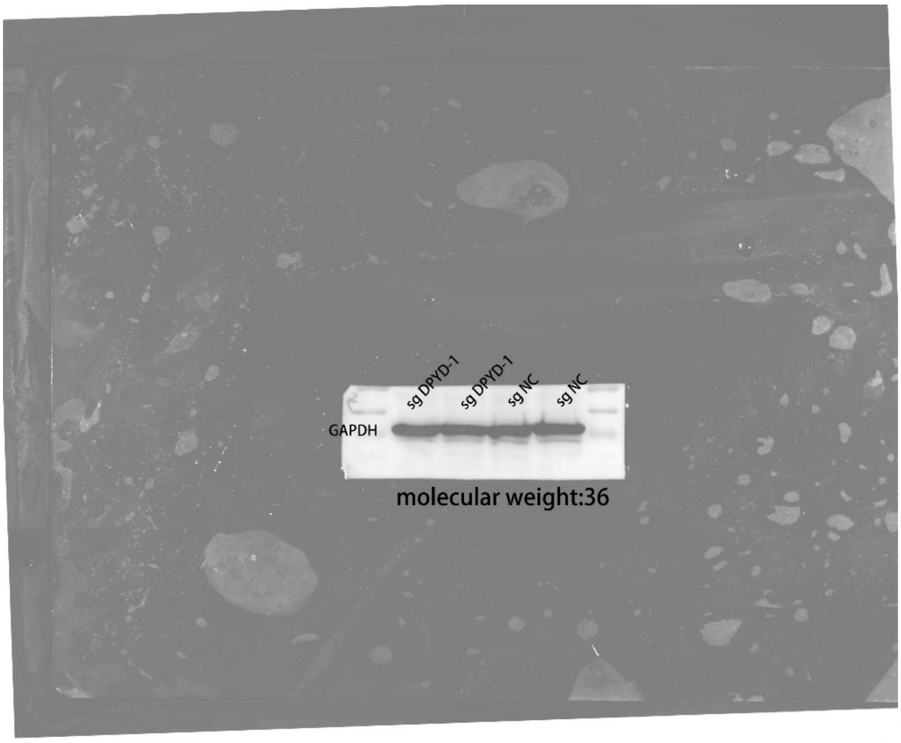
sg DPYD-1
sg DPYD-1
sg NC
sg NC
GAPDH
molecular weight:36

sg DPYD2-1

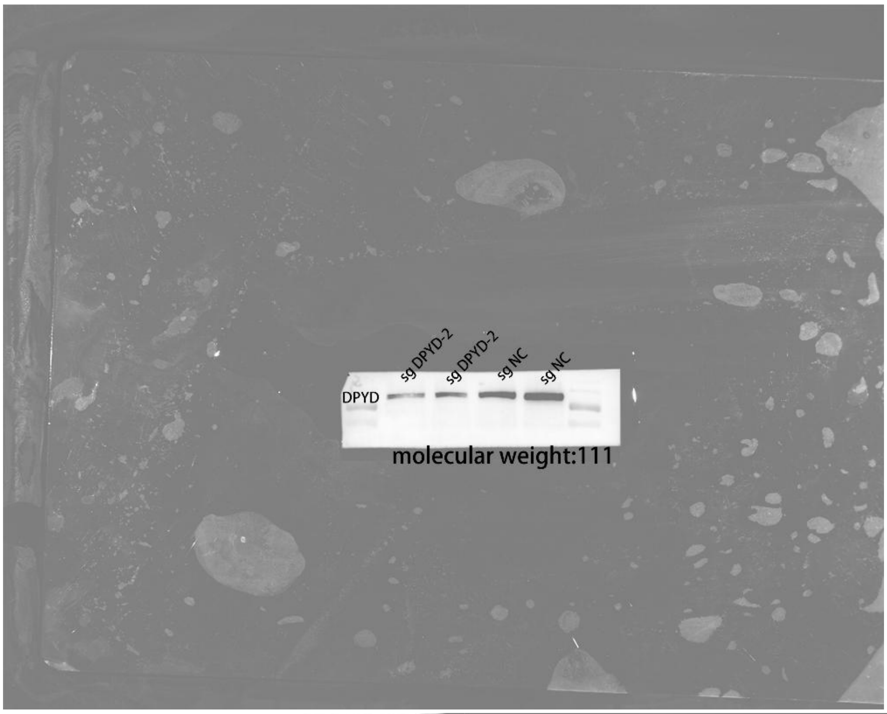
sg DPYD-2
sg DPYD-2
sg NC
sg NC
DPYD
molecular weight:111

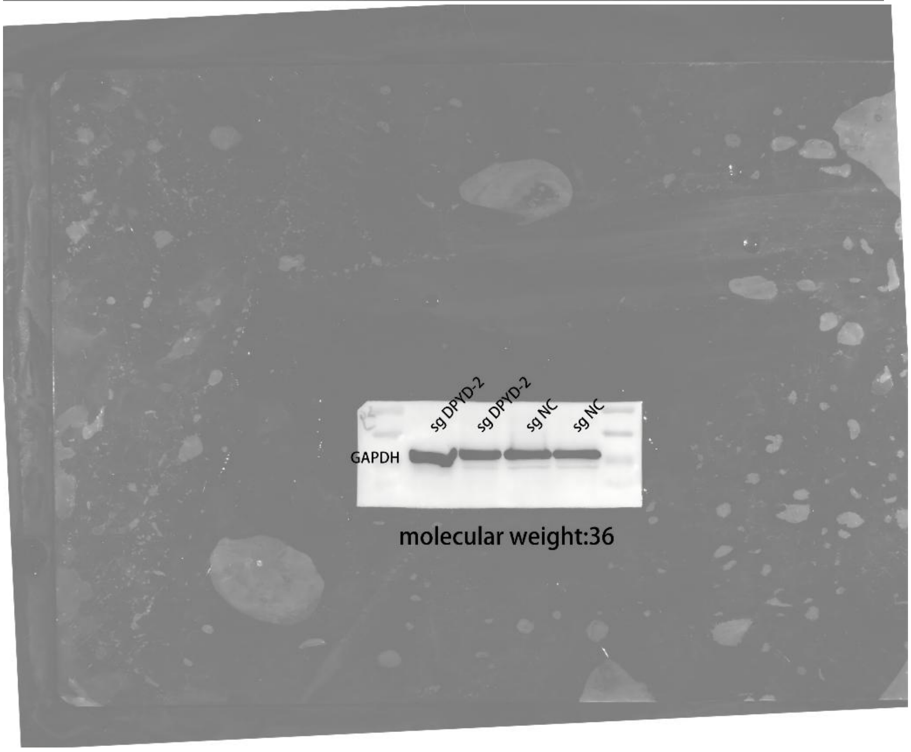
sg DPYD-2
sg DPYD-2
sg NC
sg NC
GAPDH
molecular weight:36

Sg DPYD

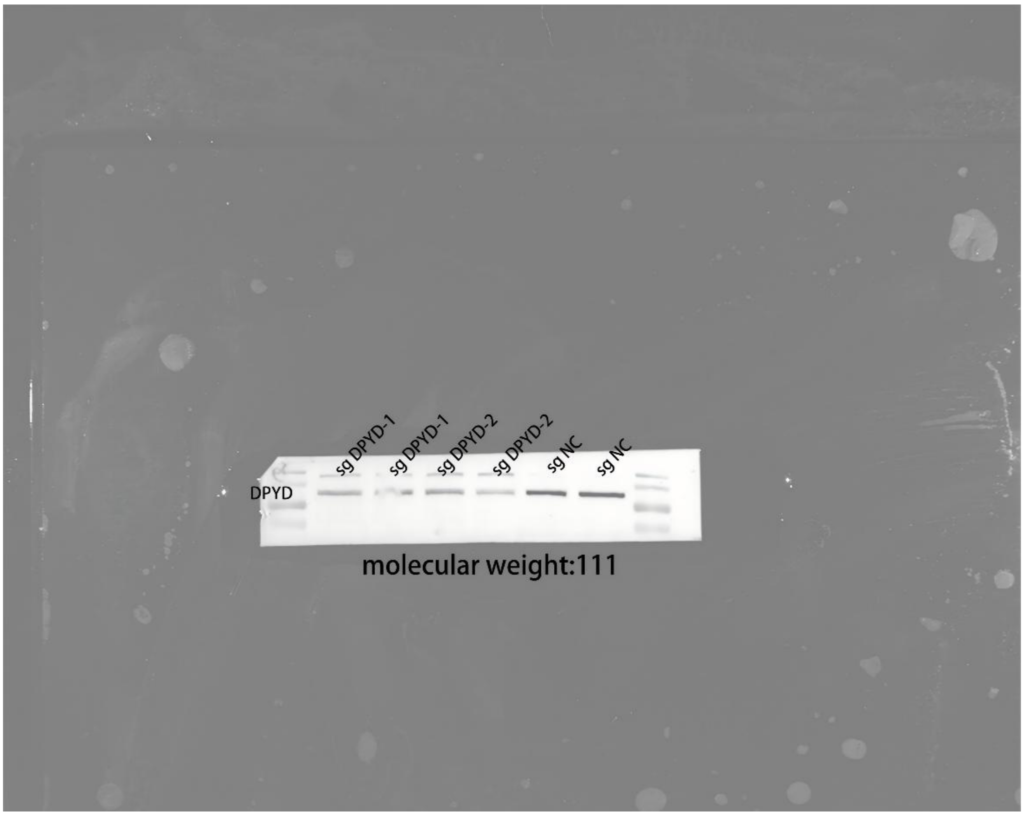
sg DPYD-1
sg DPYD-1
sg DPYD-2
sg DPYD-2
sg NC
sg NC
DPYD
molecular weight:111

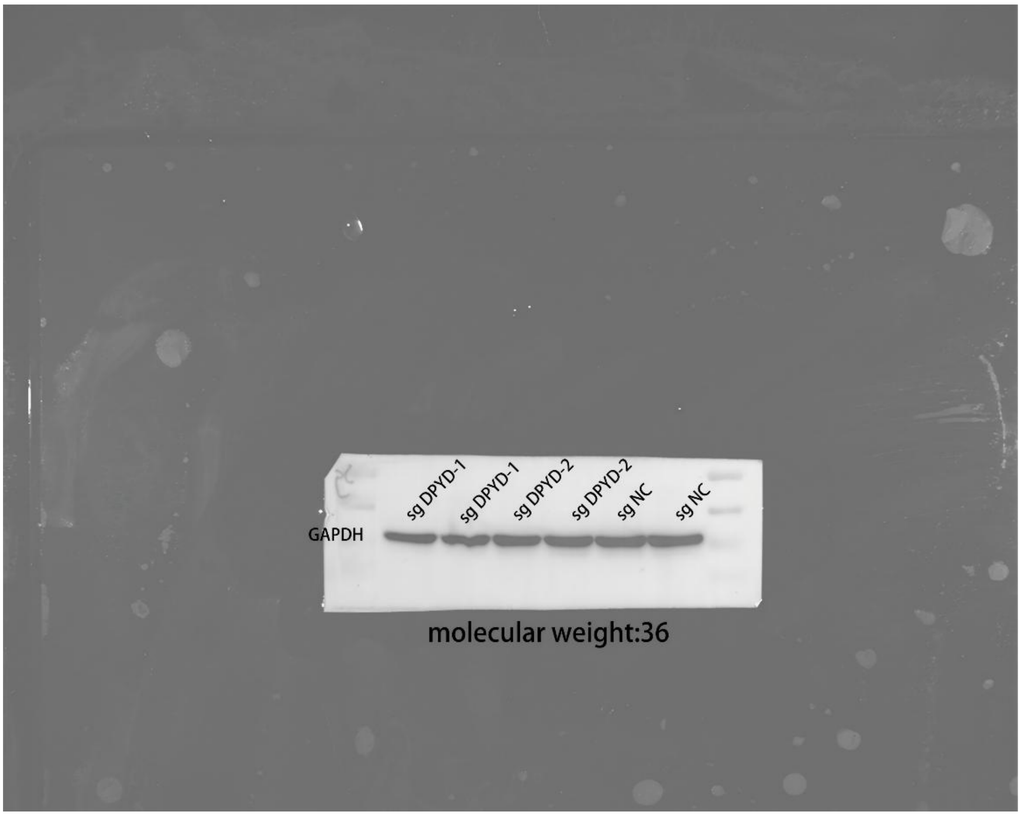
sg DPYD-1
sg DPYD-1
sg DPYD-2
sg DPYD-2
sg NC
sg NC
GAPDH
molecular weight:36

Figure 12 D

OE DPYD1

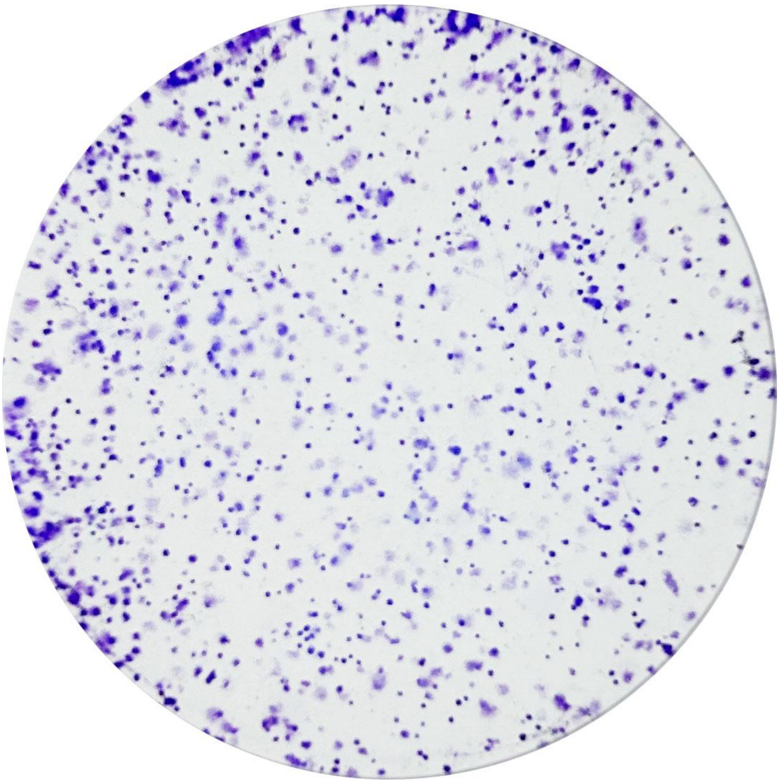

OE DPYD2

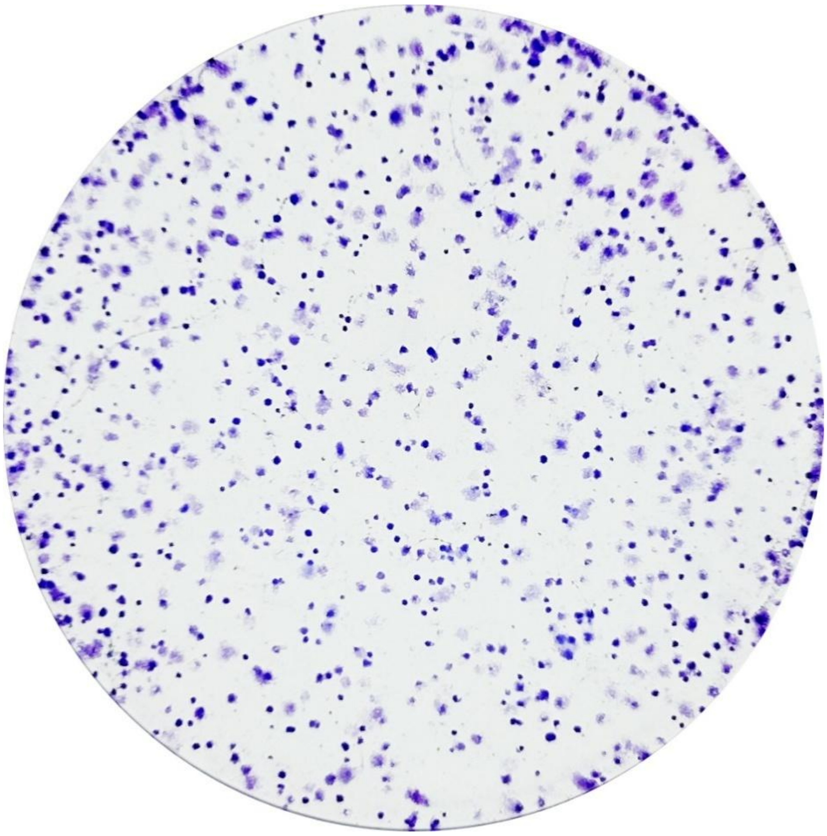

OE DPYD3

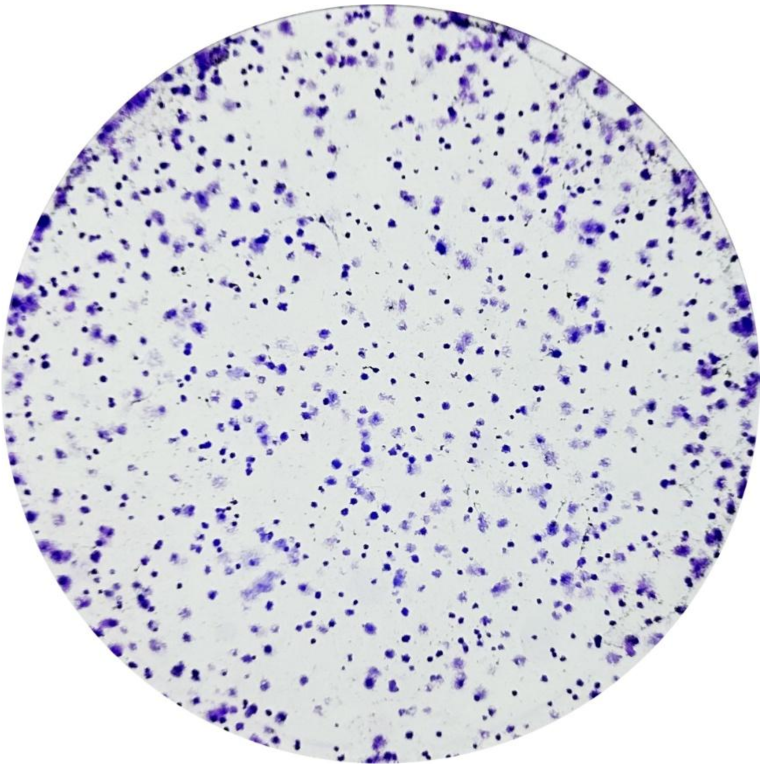

OE NC

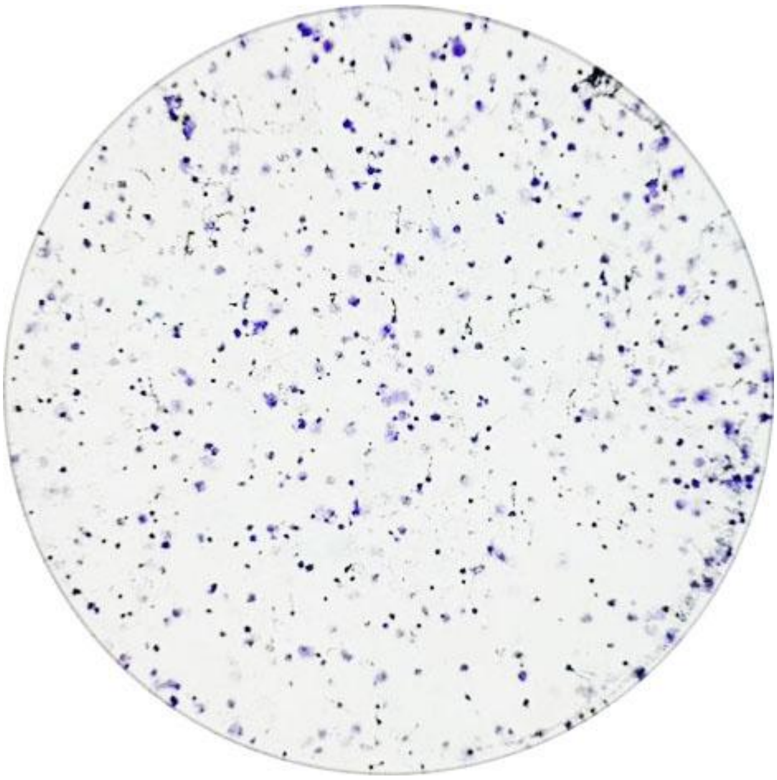

sg DPYD1.1

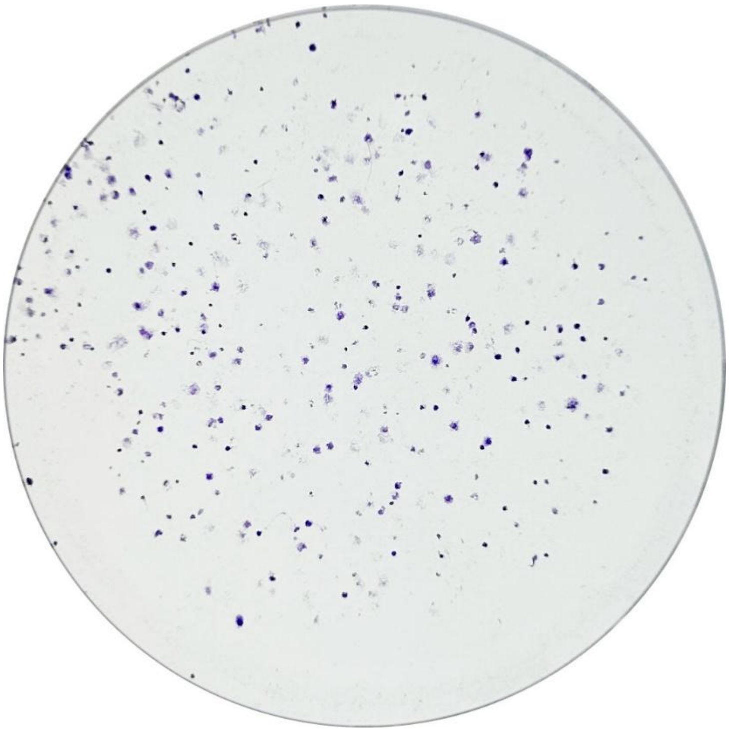

sg DPYD1.2

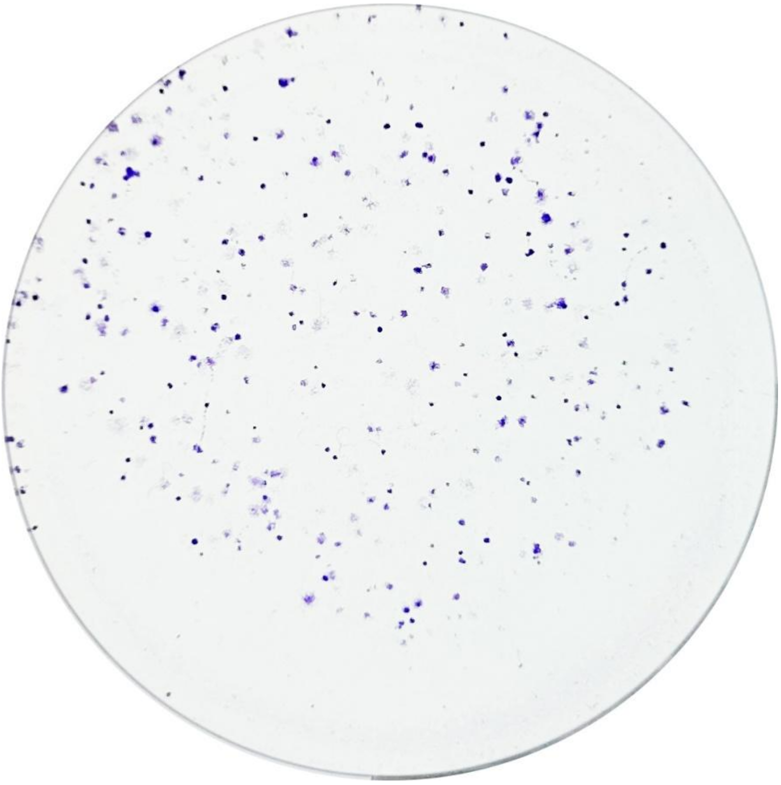

sg DPYD1.3

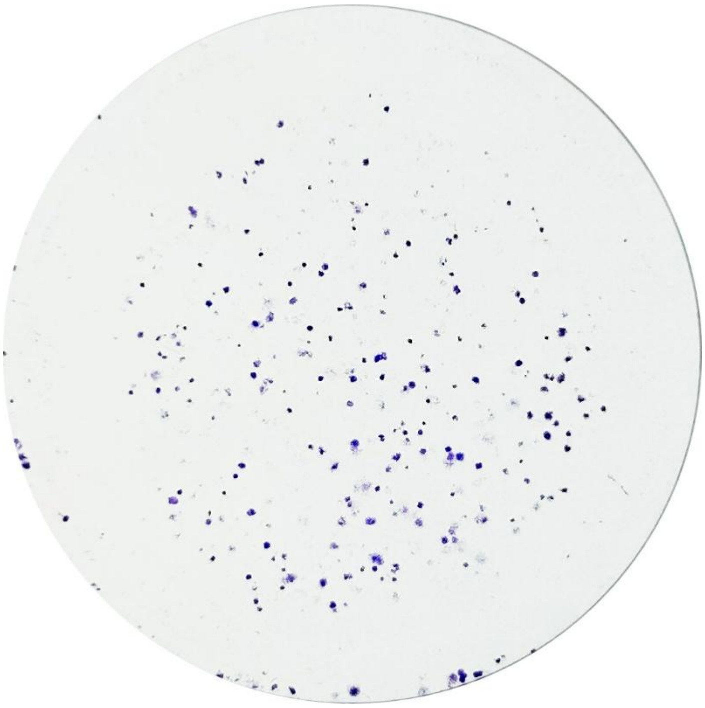

sg DPYD2.1

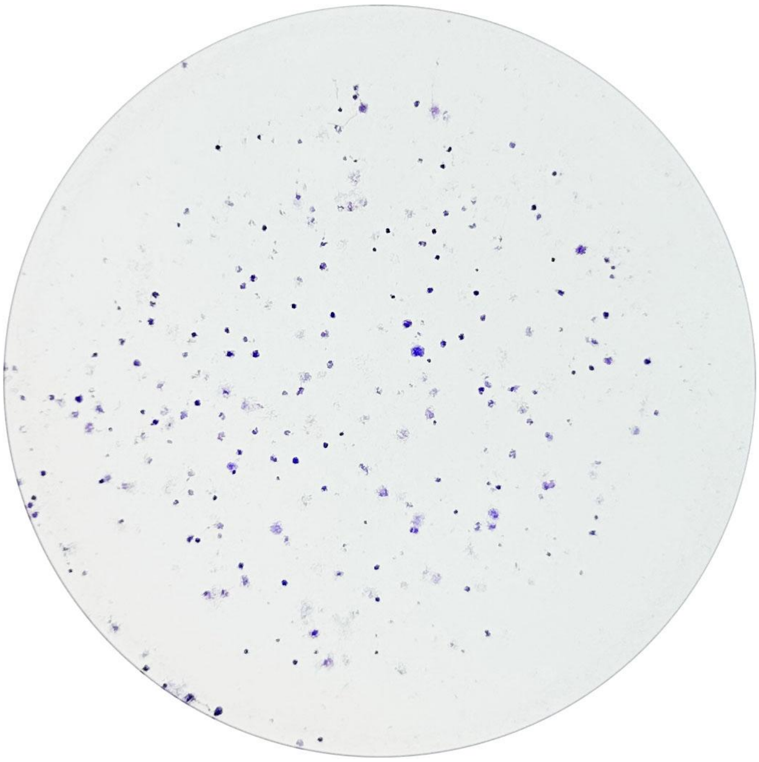

sg DPYD2.2

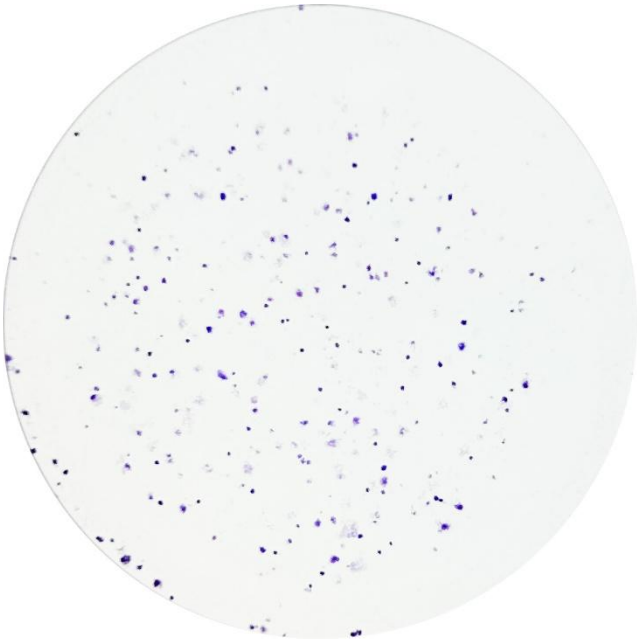

sg DPYD2.3

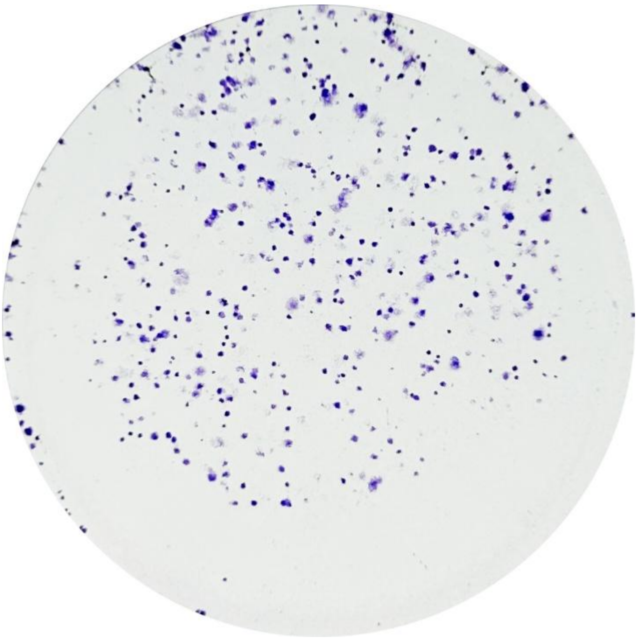

Sg NC、

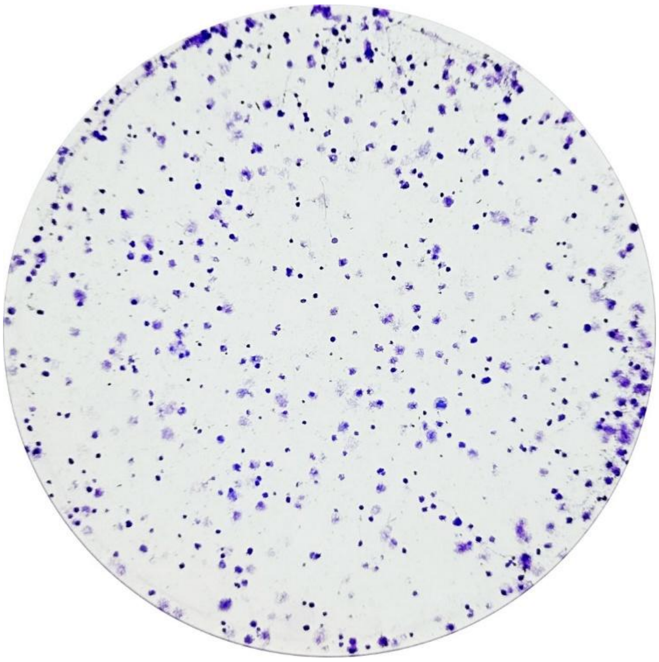

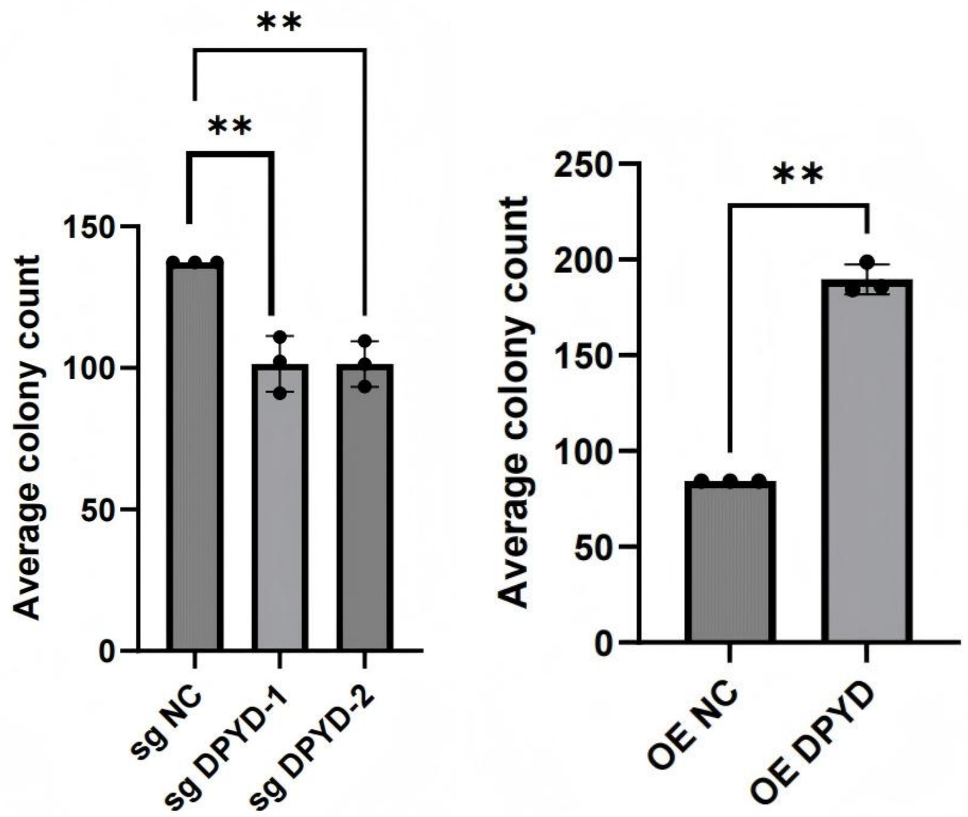

**
**
150
100
50
0
Average colony count
sg NC
sg DPYD-1
sg DPYD-2
250
200
150
100
50
0
**
Average colony count
OE NC
OE DPYD

Figure 12 E

BXPC3　con1 0h

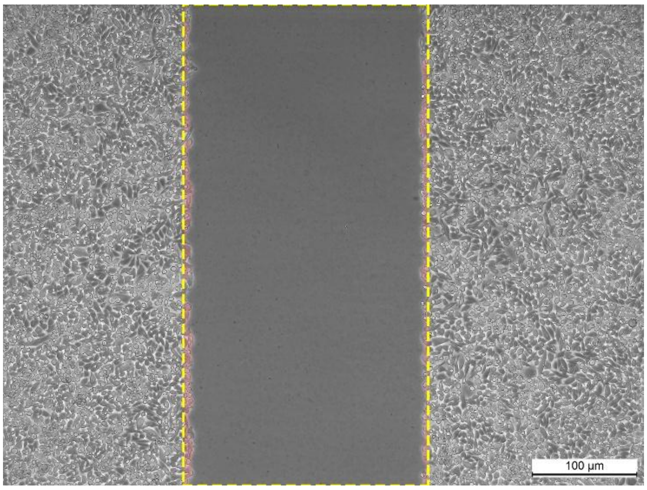

BXPC3　con1 48h

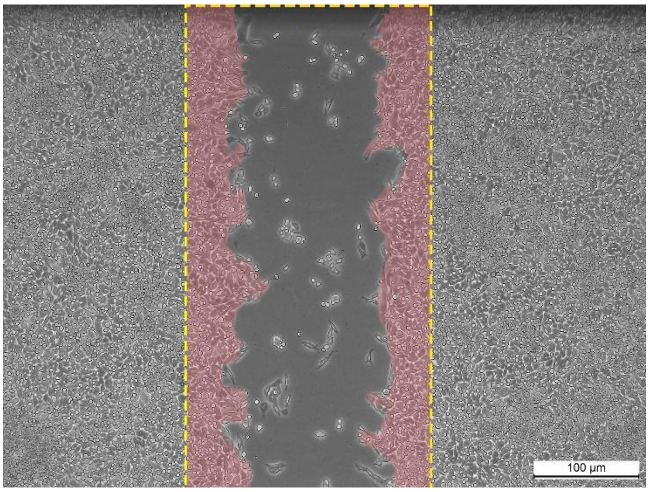

BXPC3　con2 0h

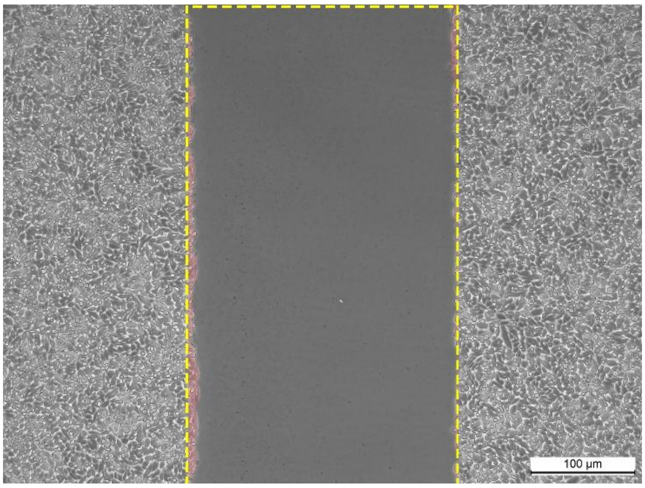

BXPC3　con2 48h

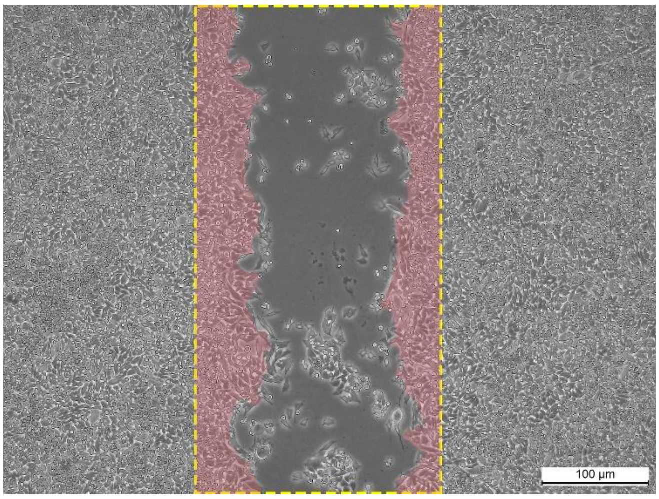

BXPC3　con3 0h

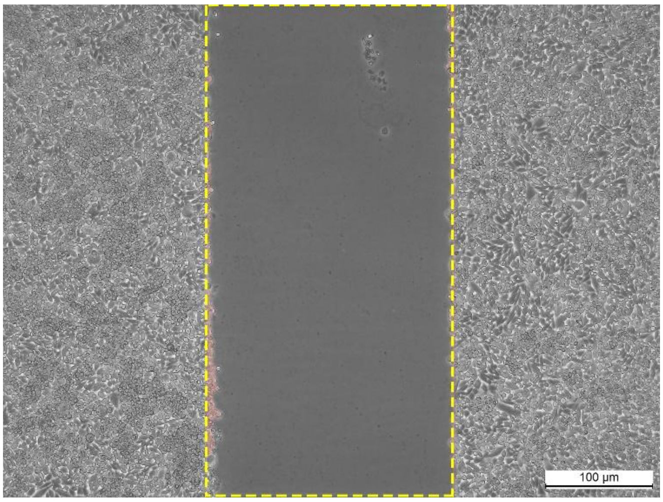

BXPC3　con3 48h

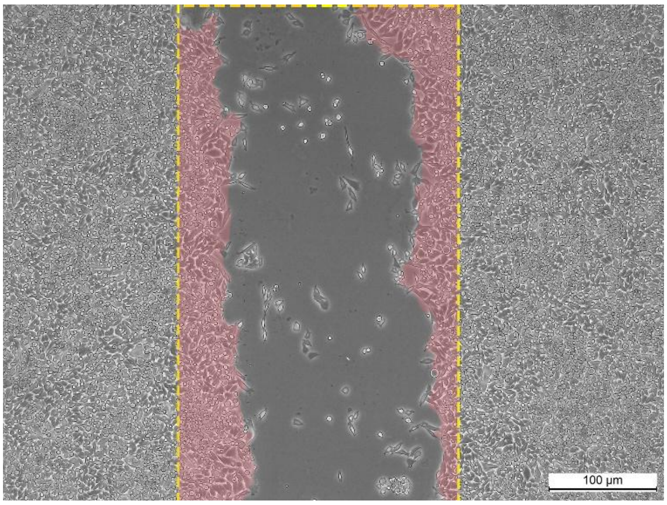

BXPC3 sg1.1 0h

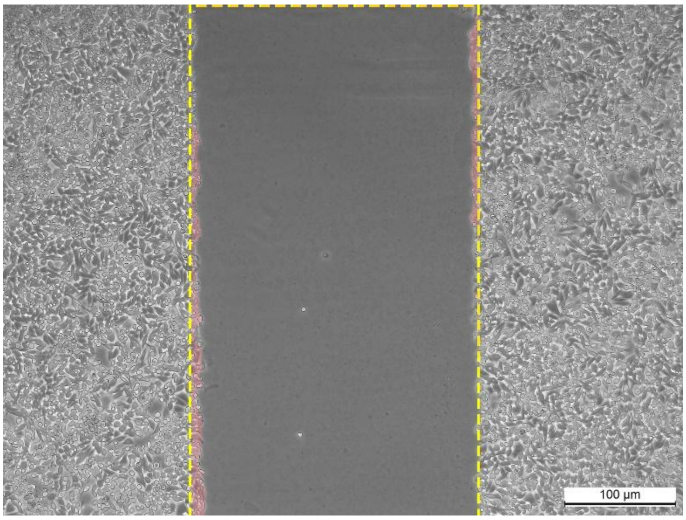

BXPC3 sg1.1 48h

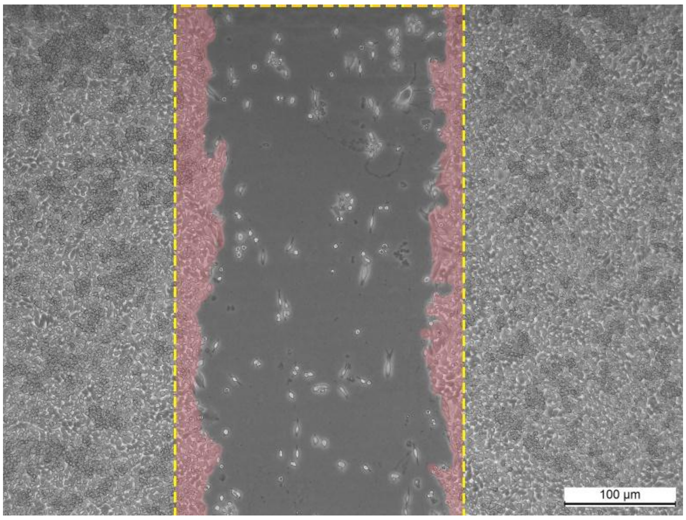

BXPC3 sg1.2 0h

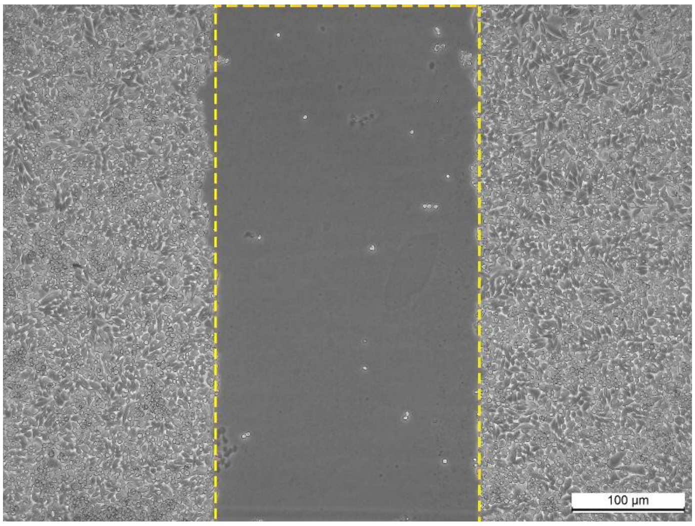

BXPC3 sg1.3 48h

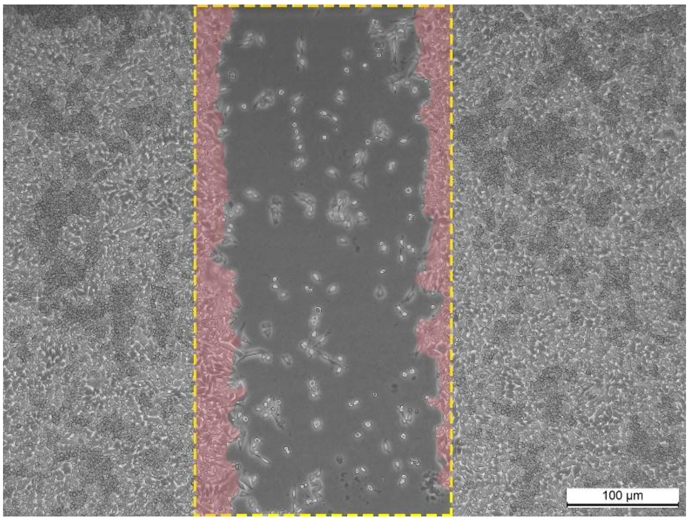

BXPC3 sg1.3 0h

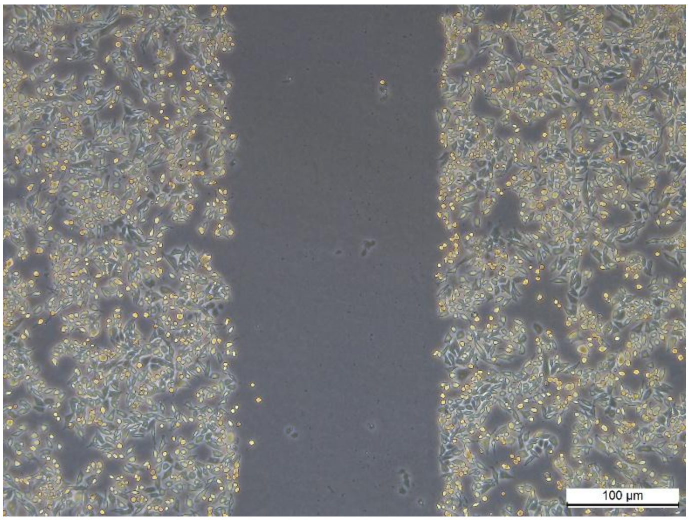

BXPC3 sg1.3 48h

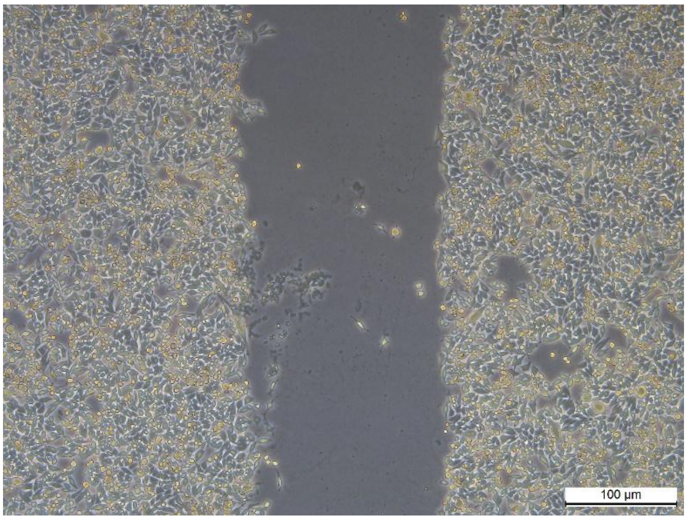

BXPC3 sg2.1 0h

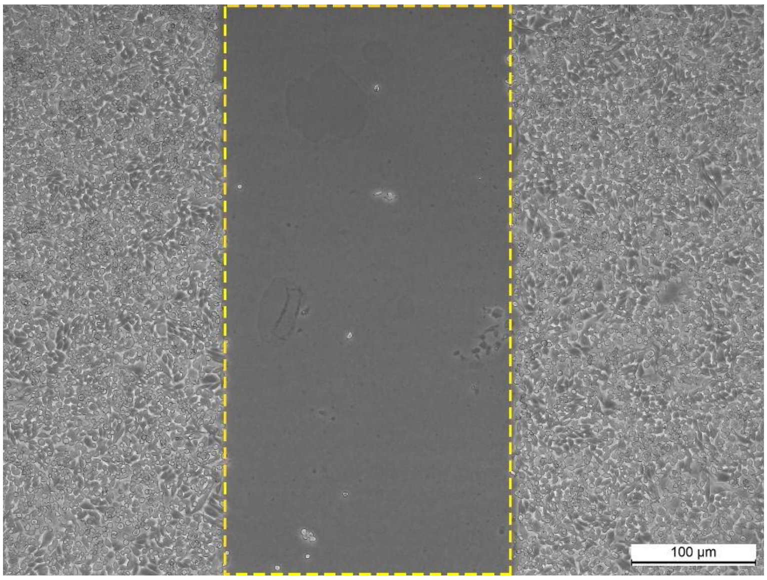

BXPC3 sg2.1 48h

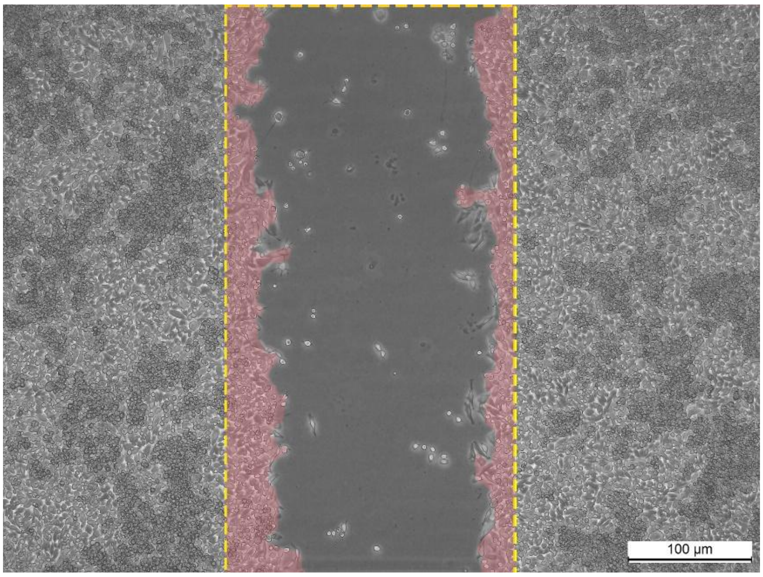

BXPC3 sg2.2 0h

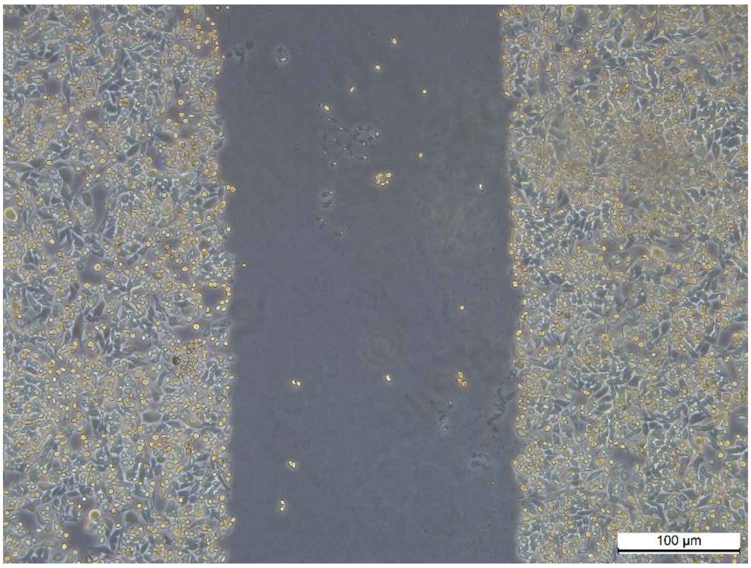

BXPC3   sg2.2 48h

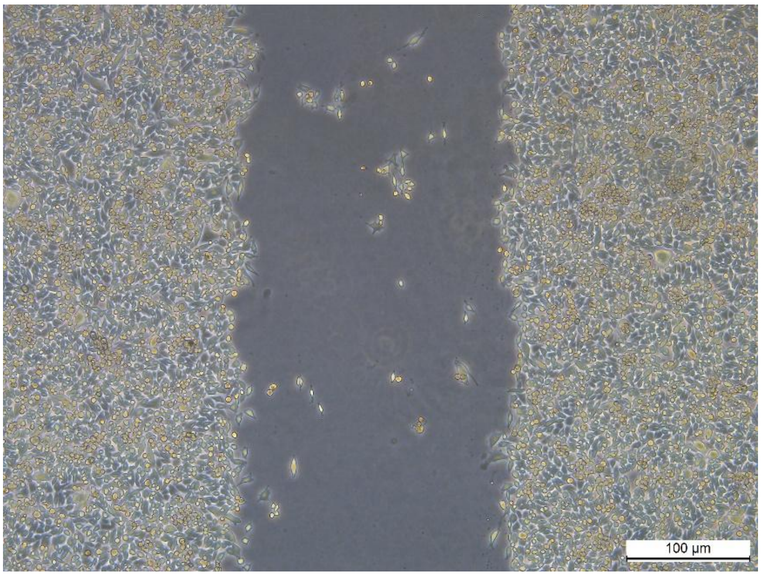

BXPC3   sg2.3 0h

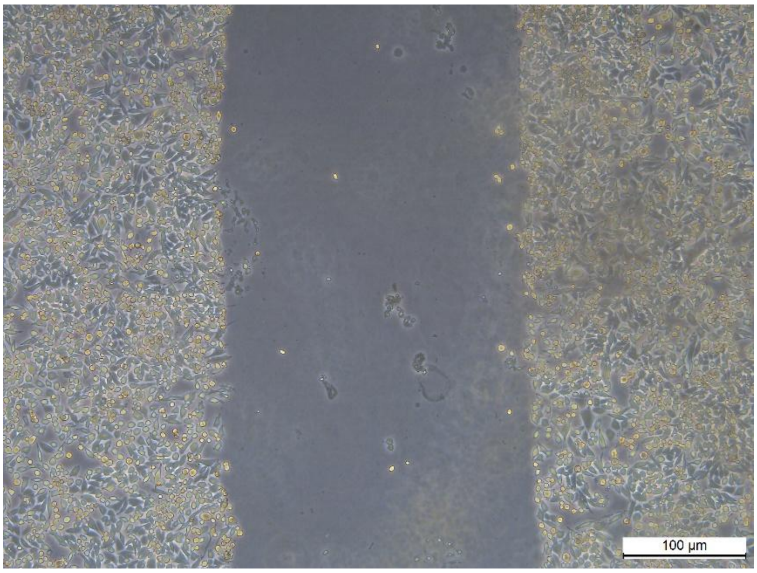

BXPC3   sg2.3 48h

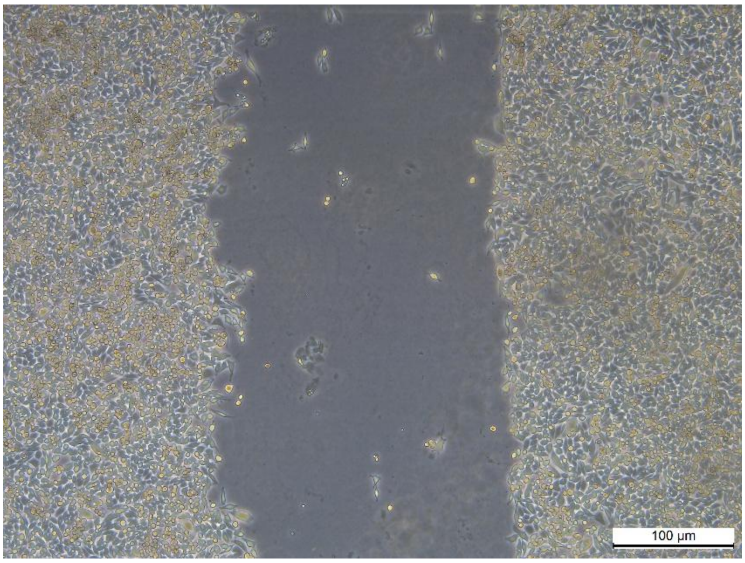

BXPC3　OE1.1 0h

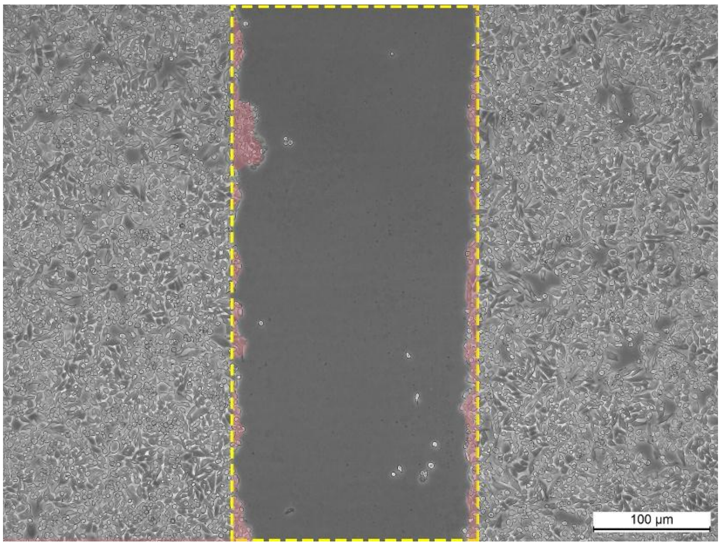

BXPC3　OE1.1 48h

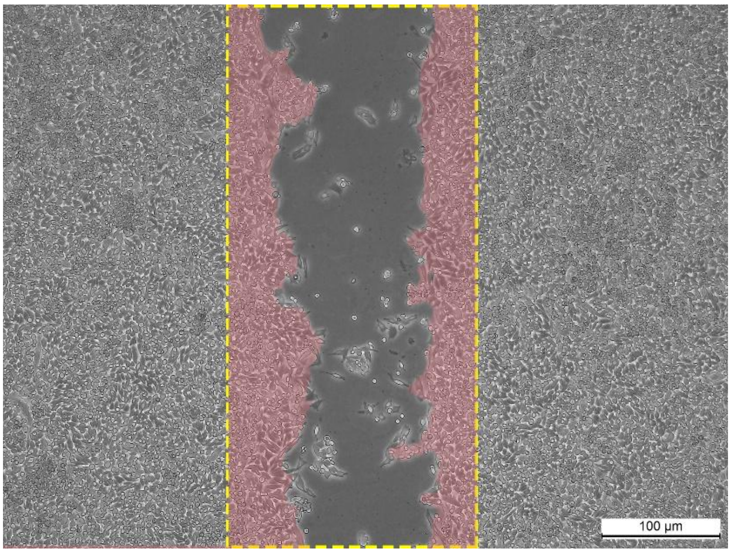

BXPC3　OE1.2 0h

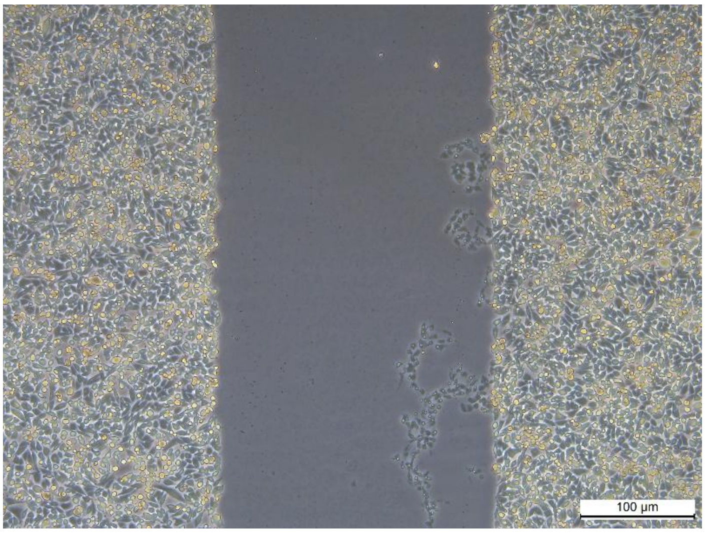

BXPC3 OE1.2 48h

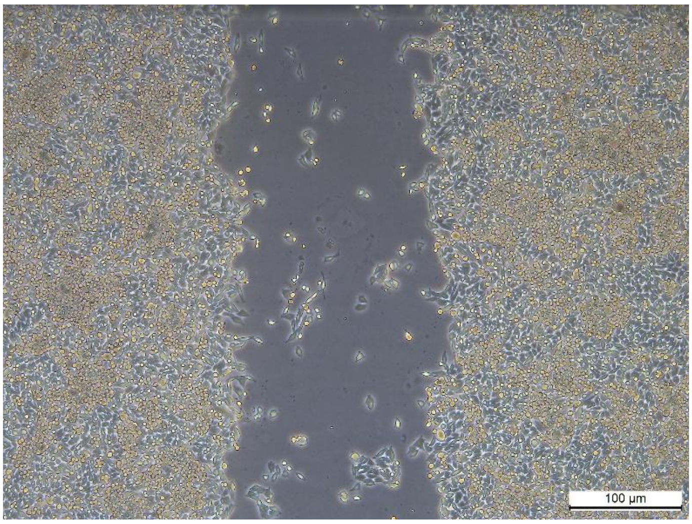

BXPC3 OE1.3 0h

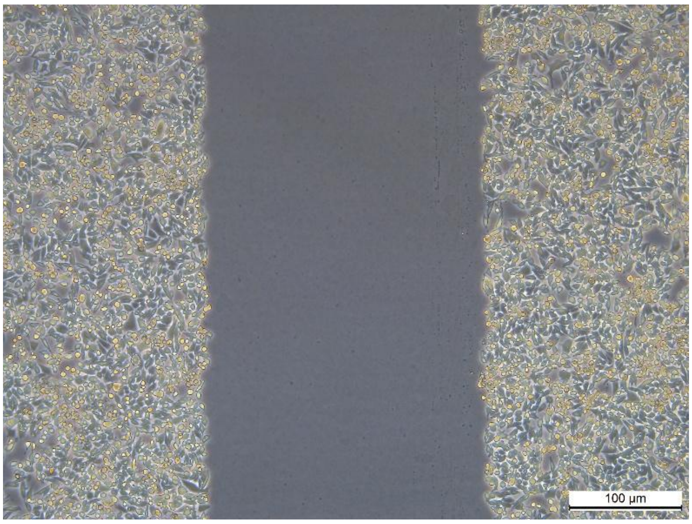

BXPC3 OE1.3 48h

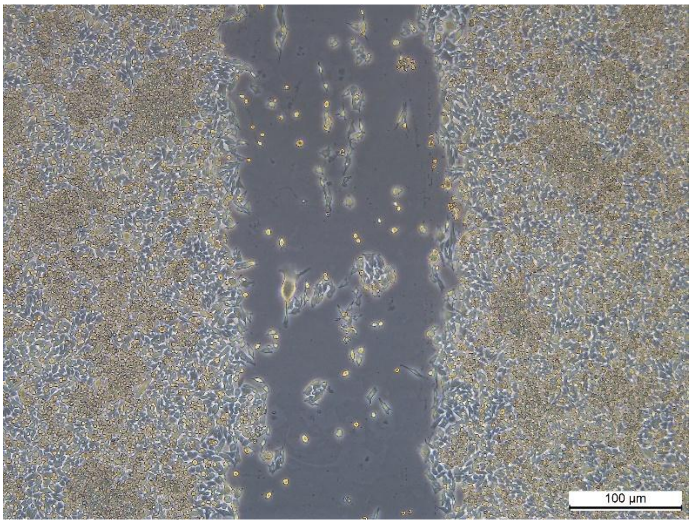

BXPC3　OE NC1.1 0h

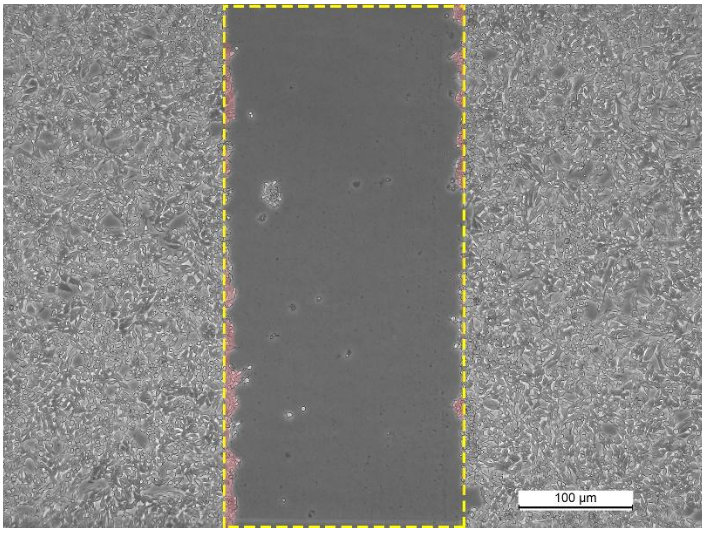

BXPC3　OE NC1.1 48h

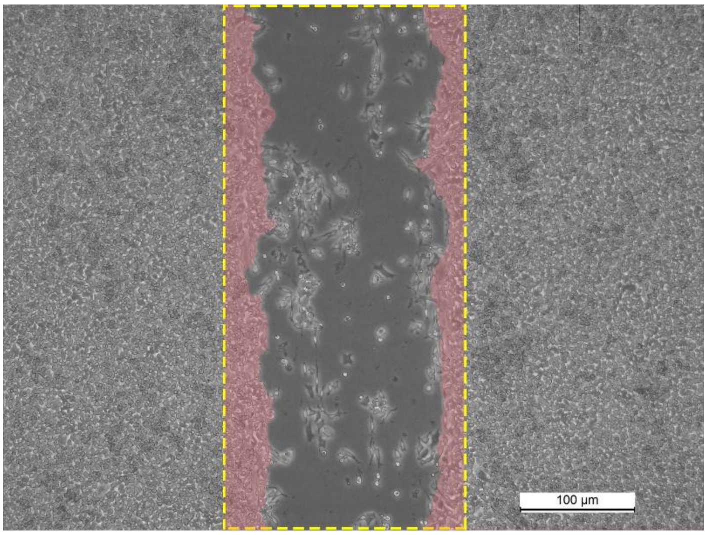

BXPC3　OE NC1.2 0h

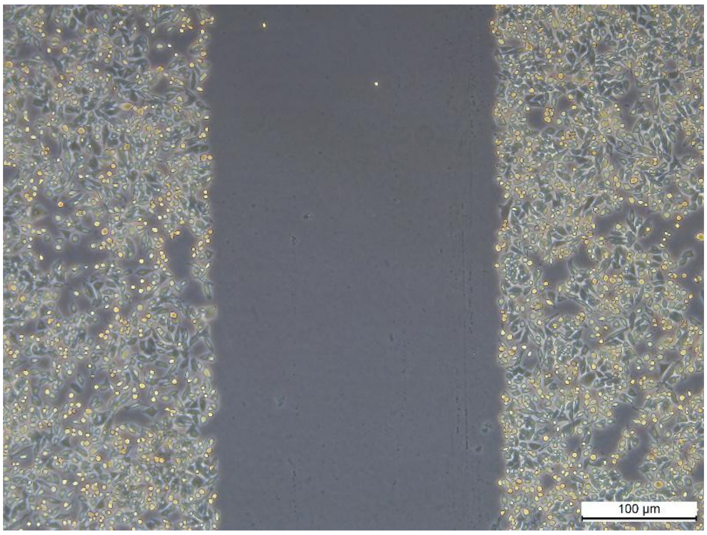

BXPC3　OE NC1.2 48h

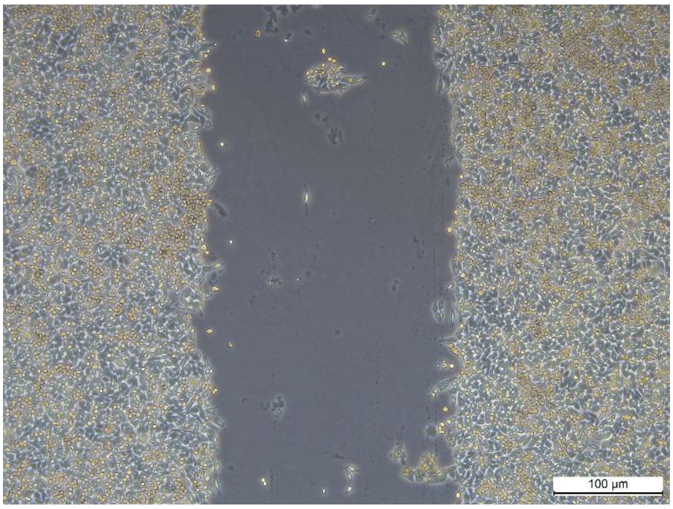

BXPC3　OE NC1.3 0h

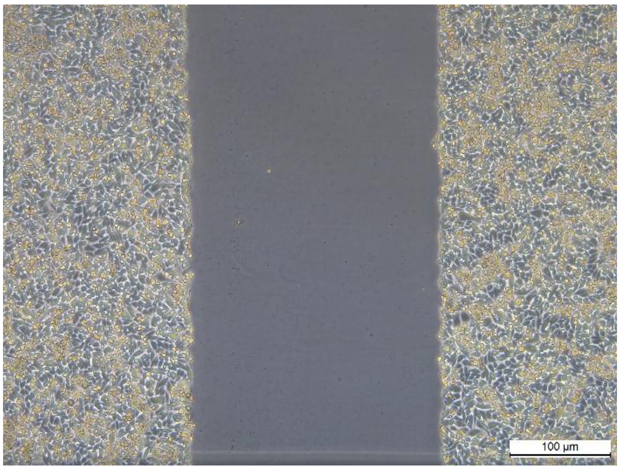

BXPC3　OE NC1.3 48h

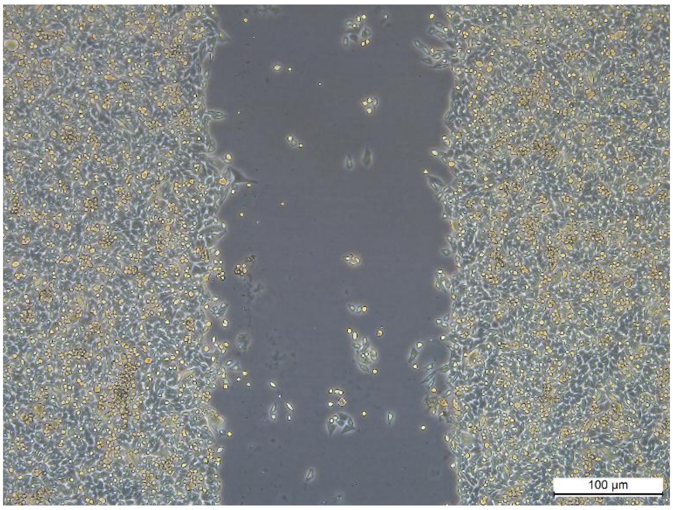

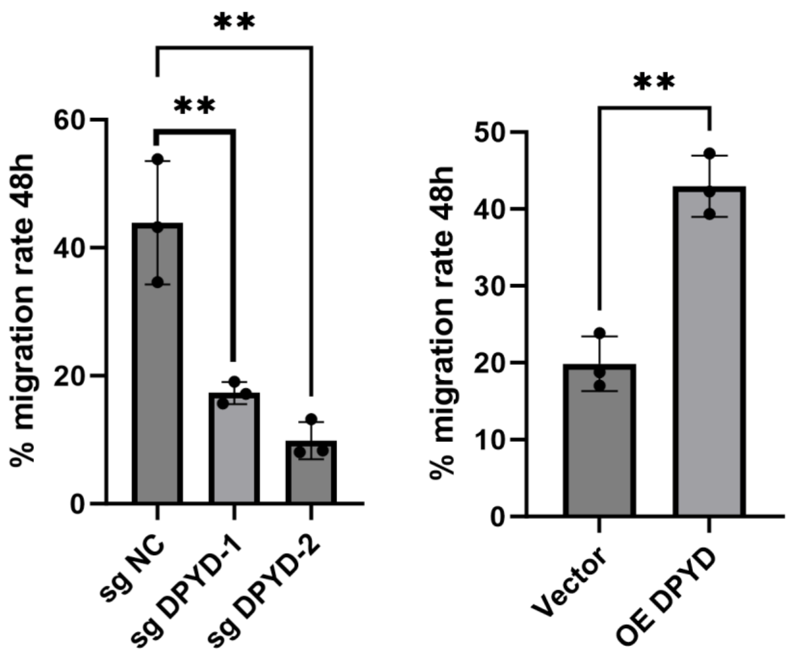

**
**
60
40
20
0
% migration rate 48h
sg NC
sg DPYD-1
sg DPYD-2
**
50
40
30
20
10
0
% migration rate 48h
Vector
OE DPYD
