# Supplementary figures and images for: Integrated analysis of metabolic gene features and the immune microenvironment: identification of DPYD-mediated prognostic model and therapeutic targets in pancreatic cancer
Source: BMC Gastroenterol. 2026 Mar 14;26:245. doi: 10.1186/s12876-026-04726-4 (PMC13101390; doi:10.1186/s12876-026-04726-4)

Figure 12B  
OE-DPYD

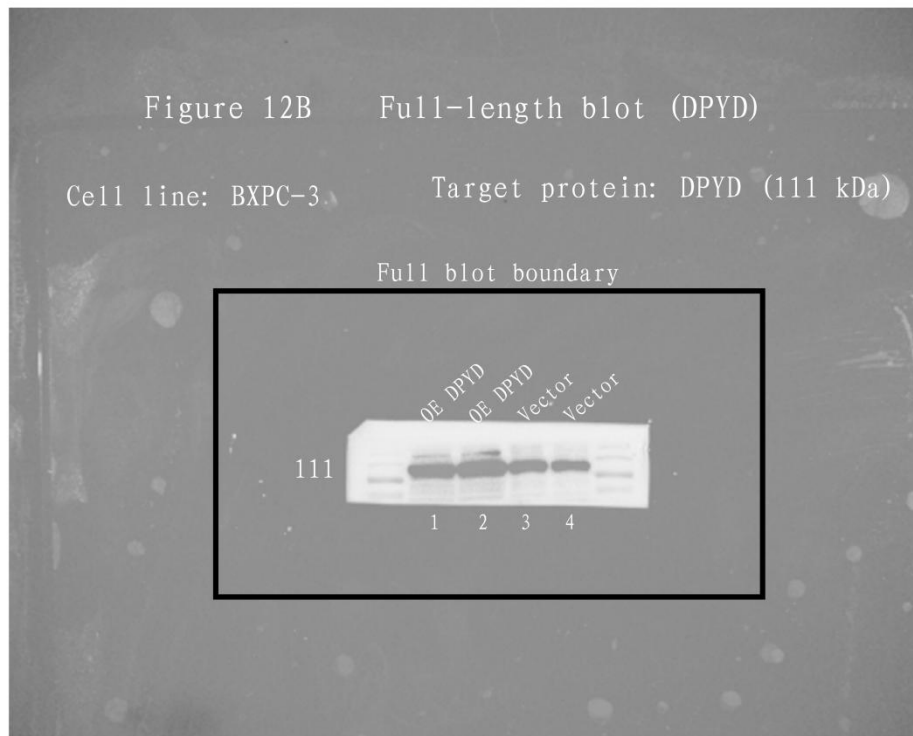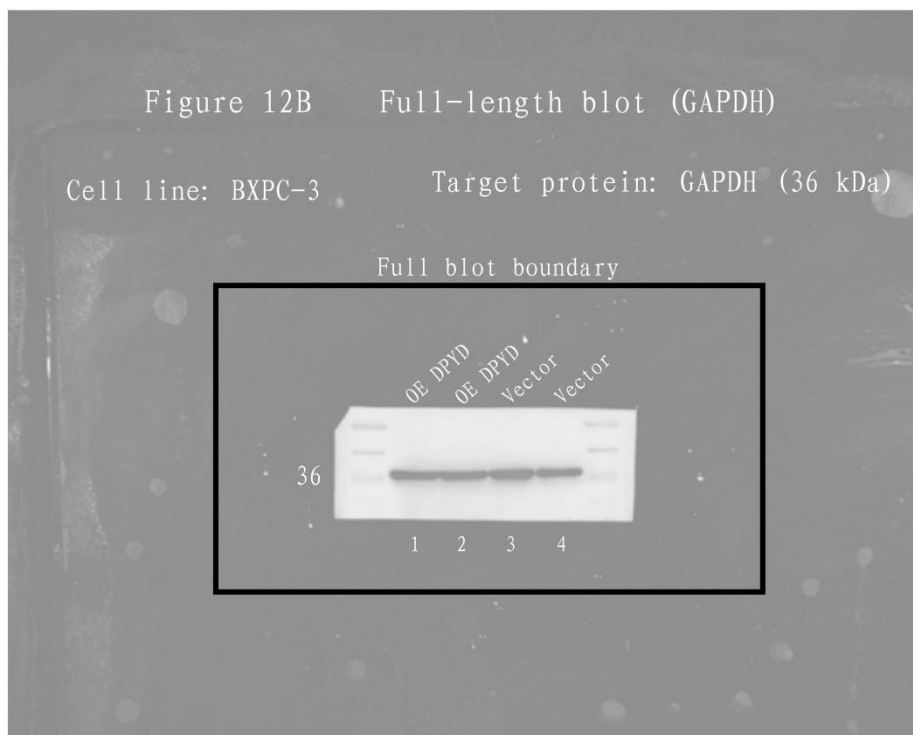

Figure 12B  
Sg-DPYD

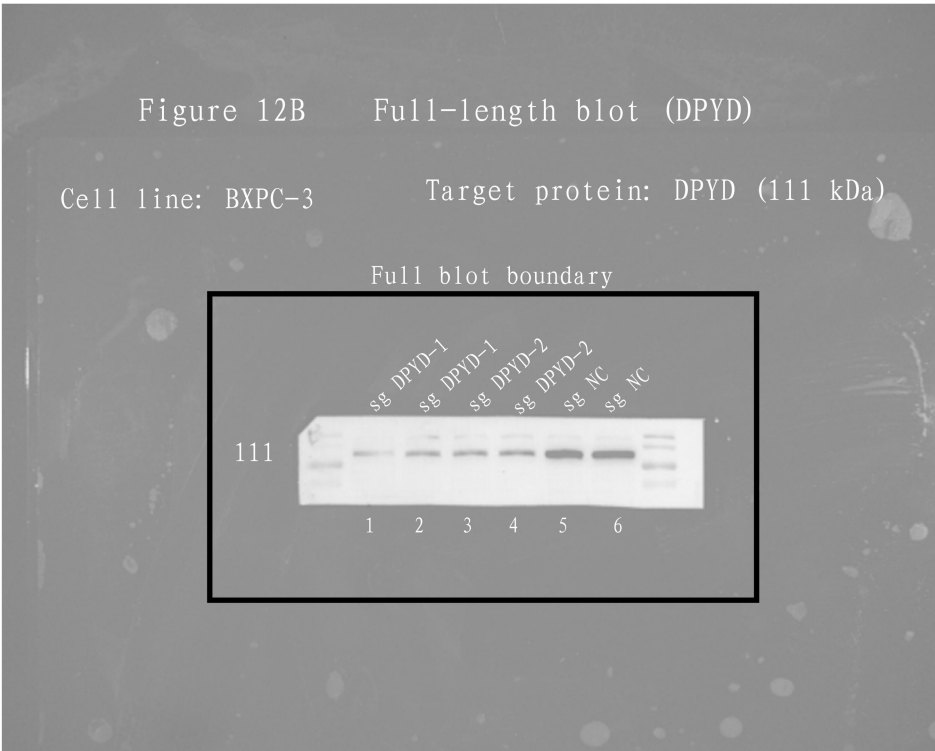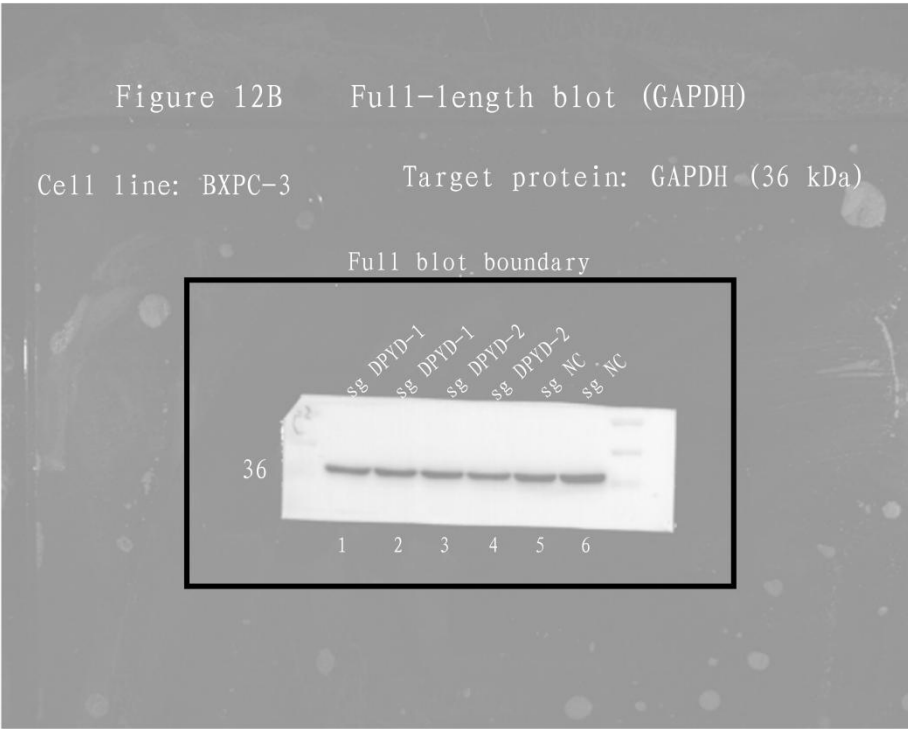

Supplement: Supplementary file 14 — Supplementary Material 14. [file 12876_2026_4726_MOESM14_ESM.pdf]
